# Longitudinal gut microbiota dynamics in Antarctic research mission crews

**DOI:** 10.3389/fmicb.2025.1593617

**Published:** 2025-05-22

**Authors:** Min-Jung Lee, Seung-Hwan Lee, Huitae Min, Tae-Wook Nam, Soon Gyu Hong, Bumjo Oh, Joo Hyeong Kim, Yeon-Ran Kim, Bong-Soo Kim, Yeong-Jae Seok

**Affiliations:** ^1^Department of Life Science, Multidisciplinary Genome Institute, Hallym University, Chuncheon, Republic of Korea; ^2^School of Biological Sciences and Institute of Microbiology, Seoul National University, Seoul, Republic of Korea; ^3^Natural Product Research Center, Korea Institute of Science and Technology, Gangneung, Republic of Korea; ^4^MightyBugs, Inc., Busan, Republic of Korea; ^5^Division of Life Science, Korea Polar Research Institute, Incheon, Republic of Korea; ^6^Department of Family Medicine, Seoul Metropolitan Government Seoul National University Boramae Medical Center, Seoul, Republic of Korea; ^7^Gachon University Gill Hospital, Incheon, Republic of Korea; ^8^Department of Nutritional Science and Food Management, Ewha Womans University, Seoul, Republic of Korea

**Keywords:** gut microbiota, Antarctica, extreme environment, variability, cohabitation

## Abstract

**Introduction:**

A prolonged stay at Antarctic research stations poses unique challenges due to extreme environmental conditions, restricted diets, and cold temperatures, all of which can influence the gut microbiota, an important factor in host health. However, our understanding of how the Antarctic environment affects the gut microbiota remains limited due to small cohort sizes and short study durations.

**Methods:**

We analyzed 467 fecal samples collected longitudinally from 48 participants who stayed at Antarctic stations for up to 16 months.

**Results:**

Before departing to the Antarctic bases, male participants exhibited three distinct types of gut microbiota, which were differentially altered during and after the stay, depending on the pre-existing microbiota type. Prevotella-dominant microbiota was more susceptible to environmental changes, including the diet, compared with Bacteroides-dominant microbiota. Although the dominant genera in the gut microbiota were stable across all microbiota types, minor genera with high variability could mediate changes in the microbiota. Sharing diets and having frequent contact resulted in cohabitation effects among genetically unrelated participants in the extremely isolated Antarctic environment. Although taxonomic composition shifted in response to the Antarctic environment, predicted functions of the gut microbiota remained relatively stable.

**Discussion:**

This study reveals that long-term residence in Antarctic research stations alters the gut microbiota in ways that depends on the intrinsic microbiota prior to the mission. These findings enhance our understanding of human gut microbiota adaptation under extreme and isolated environmental conditions.

## 1 Introduction

Researchers working in Antarctica face various psychophysiological stressors, including extremely low temperatures, blizzards, intense UV radiation, high humidity and salinity, isolation, and depletion of fresh fruits and vegetables during their journey and stay at Antarctic research stations (Moraes et al., 2020; Oldenburg et al., 2013; Rydstedt and Lundh, 2010). These extreme conditions can cause health problems including seasickness, fear, gastritis, appetite loss, and sleep disturbances, in comparison to mainland workers (Oldenburg et al., 2013). Stress from extreme environments can influence the human microbiome (Kinross et al., 2011), causing physiological changes and vice versa under various scenarios. The significance of microbiome in human health has been reported in several studies (Zheng et al., 2020; Lloyd-Price et al., 2017; Belizario and Napolitano, 2015). A recent systematic review by Klos et al. (2023) highlighted the vulnerability of the human microbiome to alterations under isolated, confined, and controlled environments (e.g., space analog) and isolated and confined environments (e.g., Antarctic station). These alterations are commonly characterized by reduced microbial diversity and shifts in dominant taxa, which can link to host immune and metabolic functions. Therefore, understanding microbiomes exposed to extreme environments is necessary to maintain the health of crews at Antarctic research stations.

The gut microbiome forms dynamic ecosystems in the gut and can be influenced by environmental factors such as geography, diet, stress, physical exercise, and body temperature (Rothschild et al., 2018; Suzuki and Worobey, 2014; Davenport et al., 2014; Karl et al., 2018b; Cook et al., 2016; Huus and Ley, 2021). These studies suggest that the gut microbiome may be modulated by extreme conditions, which could affect host health. Recent studies have underscored the relevance of the gut–brain axis in understanding microbiome dynamics under extreme conditions (Zheng et al., 2020; Lloyd-Price et al., 2017). This bidirectional communication network links the gut and brain through neural, immune, and endocrine pathways and is modulated by microbiota-derived metabolites that can influence neuroinflammation, stress response, and behavior. Several studies have reported alterations in the gut and salivary microbiome during voyages to and stays in Antarctica (Jin et al., 2014; Cameron et al., 2023; Bhushan et al., 2019; Moraes et al., 2023). However, specific alterations of microbiota have varied among studies. For instance, gut microbiota (six members) were altered during a 3-month stay in Antarctica (Jin et al., 2014). However, significant microbiota changes were detected in salivary samples, but not in fecal samples, for five members during a trans-Antarctic winter traverse expedition (over 8 months) (Cameron et al., 2023). Similar changes in salivary microbiota were also reported in studies involving 12 Indian members during a 25-day sea voyage and 30-day stay at an Antarctic station and in seven Brazilian members during a 7-week camp (Bhushan et al., 2019; Moraes et al., 2023). These inconsistencies may be caused by differences in geography, station environment, duration of stay, and the relatively small subject numbers. To determine the Antarctic environment’s influence on gut microbiota, further analysis with more subjects and longer stay periods is recommended.

Cohabitation may affect the gut microbiota of individuals within the same living quarters over 12 months because of shared diets and frequent contact (Yatsunenko et al., 2012; Dill-McFarland et al., 2019). Although the gut microbiota varies individually, it can be altered in response to dietary changes (Arumugam et al., 2011; David et al., 2014). Previous studies have reported inconsistent results on the effects of cohabitation on the gut microbiome (Song et al., 2013; Zoetendal et al., 2001), which may be attributed to various factors that affect the gut microbiome. While cohabitation has been shown to influence gut microbiota through shared diets and frequent contact, its effects in isolated environments like Antarctic stations remain unclear. Cohabitation effects can be more pronounced among unrelated individuals who share diets for extended periods in more restricted environments.

In this study, we analyzed the influence of a prolonged stay at Antarctic research stations on the human gut microbiota using 467 longitudinal fecal samples collected from 48 male participants who stayed at Antarctic stations for durations ranging from 2 to 16 months. We analyzed the effect of cohabitation in confined environments among genetically unrelated individuals who shared similar diets and living conditions. In addition, we evaluated how the limited availability of fresh food in Antarctic settings may contribute to reduced gut microbial diversity. This study aims to provide insight into microbiome dynamics in isolated and confined environments and to identify key factors influencing microbial resilience under extreme conditions.

## 2 Materials and methods

### 2.1 Ethics statement

This study was approved by the Institutional Review Board (IRB) of Seoul National University (IRB no. 1909/002-012). All participants who enrolled in this study understood the nature of the study and provided written consent.

### 2.2 Study design and fecal sample collection

Forty-eight male participants, aged 27–59 years, were recruited from the Korean Arctic and Antarctic Research Program (KAARP) members. To ensure the inclusion of healthy subjects, medical and psychological data were collected from all participants at the beginning of the study. The members stayed at the Jang Bogo Station (−74.4° geographic latitude, 164.1° geographic longitude) in Northern Victoria Land or King Sejong Station (−62.1° geographic latitude, −58.5° geographic longitude) on King George Island. Participants were classified as either “Long-term stay” (with an average stay of over 13 months) or “Short-term stay” (with an average stay of less than 3 months) based on their duration of residence at the stations. Fecal samples were collected from participants once a month (Supplementary Figure S1). Fecal samples were collected before departure from Korea (HOME), during their stay at the Antarctic stations (BASE) for ≤ 14 months, during the ship voyage after leaving the station (SHIP) for ≤ 4 months, and finally once or twice 1–6 months after returning to Korea (RETURN). Collected fecal samples were immediately stored at −*80*°C until DNA extraction.

### 2.3 Metagenomic DNA extraction and 16S rRNA gene sequencing

Metagenomic DNA was extracted from fecal samples using the QIAGEN PowerSoil DNA extraction kit (Qiagen, Hilden, Germany) as per the manufacturer’s instructions. The V3 and V4 regions of the 16S rRNA gene were amplified with Herculase II Fusion DNA polymerase (Agilent Technologies, CA, USA). Sequencing libraries were prepared using the Nextera XT library preparation kit according to the 16S metagenomics sequencing library protocol (Illumina Inc., CA, USA). Amplicons were size-selected and purified using AMpure beads (Agencourt Bioscience, MA, USA). Amplicon library concentrations were measured using quantitative real-time PCR with qPCR quantification protocol guide (KAPA library quantification kit for Illumina sequencing platform) and the Quanti-iT PicoGreen dsDNA kit (Invitrogen, CA, USA). The equimolar library for each sample was pooled and sequenced on the Illumina MiSeq platform by Macrogen (Seoul, South Korea).

### 2.4 Microbiota analysis

Amplicon sequences were analyzed using the QIIME2 pipeline (Bolyen et al., 2019). Raw sequences were quality filtered and denoised. Paired sequence merging and chimera sequence removal were performed using DADA2. Amplicon sequence variants (ASVs) from the DADA2 results were assigned taxonomic positions of representative sequences using the BLAST classifier with the EzTaxon-e database (Yoon et al., 2017). A total of 9,019,331 reads (an average of 18,220 reads per sample) was obtained from sequence analyses.

The gut microbiota was compared between samples based on the Bray-Curtis dissimilarity index using the “*vegdist*” function in the R package “*vegan*.” Differences in beta-diversity were visualized using non-multidimensional scale (NMDS) plots and tested for inference using the permutational multivariate analysis of variance (PERMANOVA; Adonis2 from the package vegan with 999 permutations) based on the Bray-Curtis dissimilarity with the “*strata*” parameter used to account for individual auto-correlation. The intra-individual compositional variability was defined as the median of Bray-Curtis dissimilarity that was calculated between samples from the same individual. The inter-individual compositional variability was defined as the median of the Bray-Curtis dissimilarity values that were calculated for an individual against all other samples at each time point.

### 2.5 Gut microbiota type clustering

Gut microbiota variations in HOME samples were determined using Dirichlet multinomial mixture (DMM) modeling with the R package “*DirichletMultinomial*” (Holmes et al., 2012). The lowest Laplace approximation score determined the best number of partitions. The amount of constrained variation in microbiota composition was determined using the Bray-Curtis dissimilarity and was visualized with NMDS plots. The significance of parameters between the DMM clusters was calculated using the Kruskal-Wallis test.

### 2.6 EnvFit analysis

To identify the genera associated with baseline gut microbiota, we performed environmental fitting analysis. The effect size and significance of genera on microbiota type were determined using the “*envfit*” function in the R package “*vegan*,” which compared the difference in the centroids of each feature relative to the total variation. Ordination was performed using NMDS based on the Bray-Curtis dissimilarity. Significance was determined based on 999 permutations. Results with *p* < 0.05 were considered significant.

### 2.7 Similarity index analysis

To assess the temporal conservation of gut microbiota between DMM clustering during the Antarctic stay, we calculated similarity indices over time. The Bray-Curtis dissimilarity index between DMM clusters of male subjects during the stay was used to evaluate the microbiota conservation between the DMM clusters. Bray-Curtis dissimilarity was visualized against the elapsed time and applied linear regression model over time. The slope of a linear model fitted to the composition changes over time represented the rate of change in the gut microbiota.

### 2.8 Linear mixed effects model and intra-class correlation analysis

To evaluate intra- and inter-individual variation in taxonomic features assess longitudinal stability of gut taxa, we applied intra-class correlation (ICC) analysis. The relationship between intra- and inter-individual gut microbiota variation and taxonomic features was analyzed using the linear mixed-effects model with the “*lmer*” function in the R package “*lme4*” (Bates et al., 2015). Median intra- and inter-individual Bray-Curtis dissimilarities of the gut microbiota were used as fixed effects, and the *p*-values for false discovery rate were adjusted using the *p*-value distribution. The mean and variance for each genus were modeled, using individuals as random variables, on log10-transformed counts from the subjects using mixed-effect models without the fixed effect. The total variance was partitioned into the intra- and inter-individual variances, and ICCs for both taxonomic and functional features were calculated using “*ICCest*” function in the R package “*ICC*.” The ICC estimation used variance components from a one-way analysis of variance (inter-individual variance and intra-individual variance; ICC = Var_*inter*_/[Var_*inter*_ + Var_*intra*_]). ICC values ≥ 0.75 for taxonomy and those ≥ 0.5 for functional data over time were considered stable features. The reproducibility of gut microbiota measurements was investigated across time stratified by DMM clusters, and the ICC was calculated for comparisons between “During Stay” (DS) and “After Leaving” (AL). Calculated ICC results were used to perform a linear regression model analysis to calculate the residuals for each genus. To evaluate both abundance-based and stability-based patterns, we applied two complementary criteria for classifying gut microbial taxa. Genera with a mean relative abundance < 1% across all samples were defined as rare taxa, while those over 10% were considered dominant taxa. To assess longitudinal stability, ICC values were used to classify genera as stable (ICC ≥ 0.75) or variable (ICC < 0.25). These classifications enabled comparison of taxon-specific resilience and variation across different gut microbiota types and time periods.

### 2.9 Network analysis

Dominant genera in each DMM cluster were estimated using FastSpar based on Pearson’s correlation with 1,000 bootstraps (Watts et al., 2019). Dominant genera with a median abundance ≥ 0.1% and prevalence ≥ 30% in each DMM cluster were included in the analysis. Pseudo *p*-values were computed by determining the proportion of simulated bootstrapped datasets exhibiting a correlation at least as extreme as the one calculated for the original dataset. The correlation network was visualized using the R package “*qgraph*.” Significant correlations (*p* < 0.05) are shown in the network. Node sizes were scaled on the node centrality measure, which was determined using the R package “*igraph*.” Edge thickness denoted a FastSpar correlation ranging from values of −0.5 to 0.5, with a correlation *p*-value < 0.05 being represented. Network hubs were identified using the PageRank algorithm, which was a link-analysis method with the underlying assumption that hubs were more connected to other nodes than non-hub nodes (Layeghifard et al., 2019). Genera with the PageRank > 0.3 were selected as keystones.

### 2.10 Longitudinal analysis

To investigate whether key genera exhibited different longitudinal trajectories across gut microbiota type, we modeled time-dependent abundance changes using spline-based permutation analysis. The differential abundance of *Prevotella* and *Bacteroides* was examined for longitudinal patterns across different time points using the “*permuspliner*” function of the “*SplinectomeR*” v.0.1.0 permutation-based package. SplinectomeR was developed for longitudinal microbiome data analysis, wherein weighted local polynomials were employed to model genus abundance data over time and assess the possibility of two categories of individuals following a more different trajectory over time than would be expected from random change (Shields-Cutler et al., 2018). The “*sliding_spliner*” function of SplinectomeR divided the time axis into 100 segments, identified segments contributing significantly to intergroup differences, and displayed *p-*values for specific time intervals based on cluster smoothed splines. The longitudinal analysis of the gut microbiota changes over time was performed via grouping based on the DMM partition.

### 2.11 Comparing predicted function

Gut microbiota functions were predicted using the PICRUSt2 (Douglas et al., 2020). The ASV sequences obtained from QIIME2 were used for this analysis, and the Kyoto Encyclopedia of Genes and Genomes (KEGG) Orthology for ASVs was obtained. The copy number of the KEGG Orthology was normalized using the cumulative sum scaling method.

### 2.12 Statistical analysis

Statistical analysis was performed using the R software v.4.3.2. All statistical tests used were two-sided, unless specified otherwise. Significantly different taxonomic and functional features between the two groups were determined using the Wilcoxon-rank sum test in R software, which is applicable only when the samples independent. For repeated measures from the same individuals, statistical analysis was performed using a linear mixed-effects model with subjects as random effects and time as fixed effects. Dunn’s multiple comparison test was used to identify differences between two or more groups using the “*dunn.test*” function in the R package. *P*-values < 0.05 were considered significant. For each comparison, *p*-values were adjusted for multiplicity using the Benjamini-Hochberg false discovery rate correction (*q*-values). Results with *q* < 0.05 were considered statistically significant. ASV data were analyzed at the genus level. Change patterns in the continuous variable over time were analyzed using a linear regression model. Linear regression lines were depicted with 95% confidence intervals, and the coefficient *p*-values were noted.

## 3 Results

### 3.1 Gut microbiota was significantly altered according to habitation environments

**FIGURE 1:**
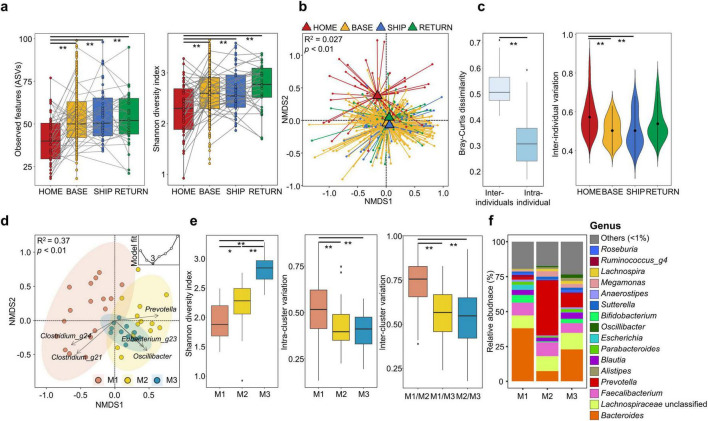
Comparing gut microbiota in different time points and analysis of compositional variation in participants at HOME. **(a)** Amplicon sequence variants (ASVs) and Shannon diversity indices of the gut microbiota from 48 participants were compared between different time points. **(b)** Microbiota composition was compared among time points using non-multidimensional scale (NMDS) plots based on Bray-Curtis dissimilarity. The *p*-values were calculated using permutational multivariated analysis of variance (PERMANOVA) using the “strata” function to account for repeated measures within individuals. **(c)** Gut microbiota variations were compared between individuals (inter-individual) or within individuals over time (intra-individual) based on Bray-Curtis dissimilarity. Inter-individual variations were compared between different time points. **(d)** Microbiota clusters determined using the Dirichlet multinomial mixtures (DMM) model with the lowest Laplace approximation indicated three clusters for gut microbiota data. Gut microbiota of male participants was clustered within three microbiota types (M1, M2, and M3) in the non-multidimensional scale (NMDS) plot (*p* < 0.01). Fiver genera had higher weights in microbiota type clustering (Spearman’s correlation *p* < 0.05). **(e)** The Shannon diversity index of the gut microbiota was compared among microbiota types. Gut microbiota variation within each microbiota type (intra-cluster variation) and between microbiota types (inter-cluster variation) was compared using box plots. The *p*-values for two group comparisons were calculated using the Wilcoxon-rank sum test. **(f)** Gut microbiota composition was compared among microbiota types at the genus level. Genera with relative abundance < 1% in each microbiota type were combined into the “others.” Bar plots show the mean relative abundance of genera in each type. HOME, before participants departed from Korea; BASE, stay at Antarctic stations; SHIP, ship voyage after leaving the stations; RETURN, after return to Korea. *p < 0.05, **p < 0.01.

The gut microbiota of 48 KAARP participants was analyzed using 467 fecal samples (Supplementary Figure S1). Gut microbiota diversity was compared across four time points (Figure 1a). The diversity increased during stay at the Antarctic stations (BASE) compared to that before their departure from Korea (HOME) and remained elevated during the ship voyage after leaving the station (SHIP) and after their return to Korea (RETURN; *p* < 0.01). These shifts were also observed in all time point analyses (Supplementary Figure S2). Individual variations were observed, and the individual change patterns (gray line in Figure 1a) differed across time points. Individual trajectories varied highlighting substantial inter-individual variability. The gut microbiota was significantly different among the four time points in NMDS plots (*p* < 0.01; Figure 1b). The inter-individual gut microbiota variations were higher than intra-individual variations across all time points. Inter-individual variations decreased from the BASE to RETURN time points (*p* < 0.01; Figure 1c). The gut microbiota exhibited temporal variations, with inter-individual differences becoming less pronounced over time, particularly during their stay at the Antarctic stations (*p* < 0.01).

### 3.2 Gut microbiota of participants before departing to Antarctica exhibited three distinct types

Gut microbiota variation was relatively high at HOME. The DMM model was used to analyze gut microbiota variation prior to their departure. Microbiota were clustered into three types based on the lowest Laplace approximation (Figure 1d). The Bray-Curtis distance revealed that the three gut microbiota types (M1, M2, and M3) exhibited significant differences (*R*^2^ = 0.37 and *p* < 0.01). DMM modeling revealed that five genera (*Clostridium*_g21, *Clostridium*_g24, *Oscillibacter*, *Eubacterium*_g23, and *Prevotella*) had higher weights (Spearman’s correlation *p* < 0.05) in microbiota clustering. The M1 type exhibited the greatest variation, whereas M3 showed the least variation. Microbiota diversity was the lowest in M1 and the highest in M3 types (*p* < 0.01; Figure 1e). The degree of gut microbiota variation within the cluster (intra-cluster variation) was significantly greater in M1 than in the other types (*p* < 0.01). In pairwise cluster comparison, the gut microbiota between M1 and M2 showed the greatest difference (*p* < 0.01; inter-cluster variation analysis) in comparison to other inter-cluster differences. *Bacteroides* was the predominant genus in the M1 type, whereas *Prevotella* was predominant in the M2 type. *Bacteroides* and *Prevotella* were co-dominant in M3 (Figure 1f).

### 3.3 Gut microbiota altered differently during the stay at the Antarctic bases depending on the microbiota type

**FIGURE 2:**
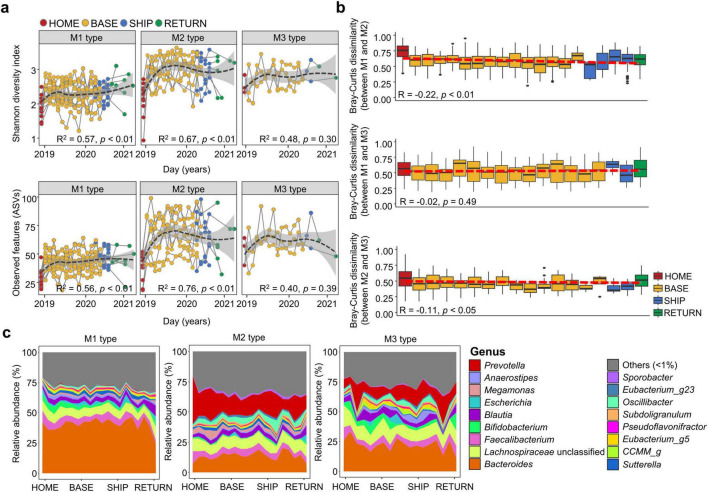
Time series changes in the gut microbiota were compared across time points. **(a)** Shannon diversity index and observed amplicon sequence variants (ASVs) in the gut microbiota of each microbiota type were compared across sampling time points. **(b)** Cohabitation effect on the gut microbiota was evaluated by the longitudinal changes of microbiota between microbiota types based on Bray-Curtis dissimilarity. The slope of a linear model fitted to the composition change over time represents the gut microbiota change rate. Gray line represents 95% confidence intervals (CIs) around the linear regression model. Coefficient and correlation *p*-values were calculated in the linear regression model. **(c)** Longitudinal changes in microbiota composition at the genus level were compared among microbiota types. Genera with relative abundance < 1% in each microbiota type were combined into the “others.” The *p*-values for two group comparisons were calculated using the Wilcoxon-rank sum test. HOME, before participants departed from Korea; BASE, stay at the Antarctic stations; SHIP, ship voyage after leaving the stations; RETURN, after return to Korea. *p < 0.05, **p < 0.01.

Gut microbiota were clustered into three distinct types at HOME. Therefore, gut microbiota alterations during the stay at the Antarctic stations were analyzed for each microbiota type. Microbiota diversity significantly increased in M1 and M2 from BASE to RETURN (*p* < 0.01; Figure 2a). However, no significant changes were identified in the M3 type over time. The significant changes in M1 and M2 were detected in participants of the long-term stay (*p* < 0.01; Supplementary Figure S3). The observed changes in participants of the short-term stay were not statistically significant in all microbiota types.

Participants stayed at two different stations in Antarctica, which could have influenced gut microbiota alterations. The gut microbiota of participants between Jang Bogo Station (JBS) and King Sejong Station (KSS) differed in the NMDS plots based on Bray-Curtis distance (*p* < 0.01; Supplementary Figure S4). However, the gut microbiota types of participants between the two stations showed a significant difference (*p* < 0.01). Therefore, observed differences in gut microbiota between the two stations can be attributed to differences in gut microbiota types at HOME.

The dissimilarity between microbiota types during the stay was analyzed to identify cohabitation effects among participants (Figure 2b). Cohabitation effects were observed, which is consistent with the decreased inter-individual variations among participants at BASE (Figure 1c). Although the dissimilarity in gut microbiota between M1 and M2 showed the most pronounced decrease after the stay in comparison to other pairwise comparisons between microbiota types (*p* < 0.01), the dissimilarity value (> 50%) was higher than that (< 50%) between M2 and M3. Alterations in the microbiota were attributed to a decrease in the relative abundance of *Prevotella*, an increase in *Bacteroides* in M2, and an increase in the relative abundance of *Prevotella* in M3 (Figure 2c). Genus composition was more similar between M2 and M3 at BASE, indicating a lower dissimilarity between them compared to other comparisons. Conversely, the relative abundance of *Bacteroides* in M1 remained elevated during BASE and subsequently declined at RETURN. The elevated relative abundance of *Bacteroides* in M2 caused an augmented similarity between M1 and M2 at BASE and SHIP. Therefore, gut microbiota exhibited disparate alterations during stay in the bases, which was dependent on the microbiota type at HOME.

### 3.4 Intra-class correlation coefficient analysis identified stable and variable genera in gut microbiota over time

**FIGURE 3:**
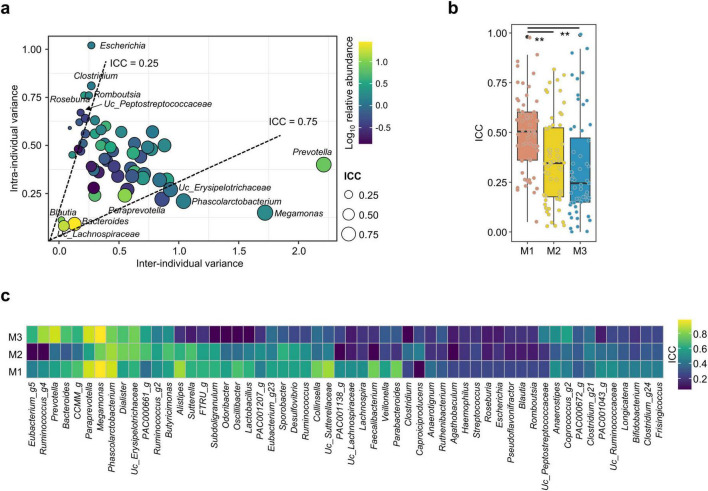
Analyzing intra-class correlation coefficients (ICC) for dominant genera in the gut microbiota. Dominant genera were selected by median abundance ≥ 0.1% and prevalence ≥ 30% in all samples. **(a)** Relationships between the inter-individual and intra-individual components of total genus variance. Dashed lines at ICC 0.25 and 0.75 separate genera with high and low intra-individual variances, respectively. Circle color indicates the relative abundance, and the circle size indicates the ICC value for each genus. **(b)** ICC values of the gut microbiota were compared among microbiota types. **(c)** ICC values for selected genera were compared among microbiota types in the heatmap plot.

The contribution of microbes to the stability of gut microbiota over time was analyzed using a linear mixed-effects model with fixed effects. In the analysis, 55 genera with a median abundance ≥ 0.1% and prevalence ≥ 30% in all samples were included (Supplementary Table S1). The ICC was calculated for selected genera to determine the influence of intra- and inter-individual variations on observed variance. The median ICC for all participants was 0.48, indicating that intra-individual variation of genera abundance was greater than the inter-individual variation. Dominant genera with high relative abundance, including *Megamonas*, *Prevotella*, *Phascolarctobacterium*, *Paraprevotella*, and unclassified (UC)_*Erysipelotrichaceae*, exhibited ICCs > 0.75, indicating stable genera throughout all time points with low intra-individual variations (Figure 3a). In contrast, genera with low relative abundance, including *Escherichia*, *Clostridium*, *Roseburia*, *Romboutsia*, and UC_*Peptostreptococcaceae*, exhibited ICCs ≤ 0.25, indicating unstable genera with high intra-individual variations. Three dominant genera, *Bacteroides*, *Blautia*, and UC_*Lachnospiraceae*, exhibited low total variances. Gut microbiota were clustered into three distinct types at HOME, and shifts in microbiota diversity and composition differed based on microbiota type (Figures 1, 2). The ICC for each genus may differ according to microbiota type. Therefore, the ICC for selected genera was calculated and compared among microbiota types.

The median ICC value in M1 (0.51) was significantly higher than that in M2 (0.35) and M3 (0.25) (*p* < 0.01; Figure 3b, Supplementary Table S2). Therefore, the gut microbiota in M1 was more stable than those in other microbiota types. ICC values for selected genera exhibited notable differences among microbiota types (Figure 3c). The number of genera with a high ICC value (≥ 0.5) was higher in M1 (29 genera) than in other types (16 and 14 genera in M2 and M3, respectively). Hence, the shift of each genus differed according to microbiota types. For instance, *Megamonas* and *Phascolarctobacterium* exhibited ICCs > 0.75 in all microbiota types, whereas the ICC value of *Prevotella* decreased below 0.59 in M1 and 0.35 in M2. These genera were stable in all participant analyses with ICCs > 0.75. However, unstable genera in all participants analyses also exhibited low ICC values (≤ 0.40) in each microbiota type. Therefore, shifts in dominant genera differed depending on microbiota type. However, the variation of rare genera (with low abundance) was commonly observed in all microbiota types.

### 3.5 Shifts of genera in the gut microbiota during stay at and after leaving the Antarctic stations differed depending on the gut microbiota type

Genera variation over time differed depending on the microbiota type. Shifts in genera observed in the gut microbiota after leaving the stations may have differed among microbiota types. Therefore, we analyzed the ICC values for each microbiota type during stay at and after leaving the stations. The HOME and BASE samples were categorized into DS and the SHIP and RETURN samples into AL. The DS group was used to identify the effect of staying at the Antarctic stations, whereas the AL group was used to identify the effect of leaving the Antarctic stations. Genera with a median abundance ≥ 0.1% and prevalence ≥ 30% in samples of each microbiota type were selected for analysis. For M1, M2, and M3, 47, 65, and 55 genera were selected, respectively (Supplementary Tables S3–S5). The median ICC value in DS was higher in M1 (0.49) than that in the other types (0.39 for M2 and 0.26 for M3). Therefore, gut microbiota in M1 were more stable than those in other types; however, longitudinal alterations of gut microbiota occurred in all participants, including those in M1. The median ICC values in AL were also higher in M1 (0.52) than those in other types (0.45 and 0.30 for M2 and M3, respectively). M1 type gut microbiota exhibited greater stability compared to M2 and M3 across all periods. Gut microbiota shifts were more dynamic in DS than in AL for all microbiota types.

**FIGURE 4:**
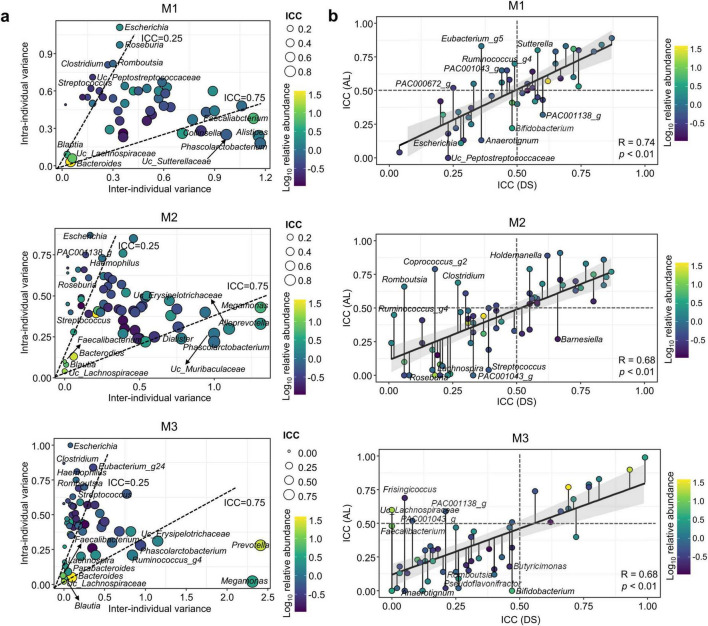
Patterns of inter- and intra-individual variations for genera in each microbiota type. **(a)** Relationships between the inter- and intra-individual components of total genus variances in each microbiota type. Dashed lines at intra-class correlation coefficients (ICCs) of 0.25 and 0.75 separate genera with high and low intra-individual variances, respectively. Circle color indicates the relative abundance, and circle size indicates the ICC value for each genus. **(b)** Changes in ICC values for each genus over time (during- and post-stay) in each microbiota type were analyzed using the linear regression model. Circle color indicates the relative abundance, and the attached line with the circle indicates the changed ICC value. The gray line represents 95% confidence intervals (CIs) around the linear model. DS, “during stay” which included HOME and BASE samples; AL, “after leaving” which included SHIP and RETURN samples; HOME, before participants departed from Korea; BASE, stay at Antarctic stations; SHIP, ship voyage after leaving the stations; RETURN, after return to Korea.

Microbiota types exhibited distinct patterns of stability in genera with ICCs > 0.75. The genera *Phascolarctobacterium*, *Alistipes*, UC_*Sutterellaceae*, *Collinsella*, and *Faecalibacterium* exhibited ICCs > 0.75 in M1, whereas *Phascolarctobacterium*, *Alloprevotella*, UC_*Muribaculaceae*, UC_*Erysipelotrichaceae*, *Dialister*, and *Megamonas* had ICCs > 0.75 in M2 (Figure 4a). *Megamonas*, *Prevotella*, *Ruminococcus*_g4, UC_*Erysipelotrichaceae*, and *Phascolarctobacterium* exhibited ICCs > 0.75 in M3. *Phascolarctobacterium* was identified as a stable genus across all microbiota types. *Bacteroides* and *Prevotella* were the predominant genera in all microbiota types at HOME. The ICC values for *Bacteroides* were ≥ 0.57 in M1 and ≥ 0.69 in M3; however, it was ≤ 0.39 in M2 (Supplementary Tables S3–S5). The ICC value for *Prevotella* was ≤ 0.44 in M2; however, it was ≥ 0.90 in M3. *Prevotella* in M1 was not included in this analysis because of its low abundance and prevalence. Therefore, longitudinal changes in dominant genera differed according to microbiota composition. In particular, the *Prevotella*-dominant microbiota type (M2) exhibited a greater longitudinal change than the *Bacteroides*-dominant microbiota type (M1). Variable genera with ICCs < 0.25 also differed among microbiota types. *Escherichia* and *Streptococcus* were identified as variable genera in all microbiota types. The number of variable genera was lower in M1 (6 genera) than that in M2 (18) and M3 (27). Genera with high relative abundance exhibited ICC > 0.75, whereas genera with low abundance exhibited ICC < 0.25 in all microbiota types. Hence, dominant genera were relatively stable compared with rare genera in the gut microbiota.

Longitudinal shifts of genera may differ between DS and AL, which could affect gut microbiota resilience. Therefore, we compared ICC values between DS and AL for each microbiota type (Figure 4b). Positive correlations of ICC values were observed in all microbiota types (*R* ≥ 0.68, *p* < 0.01). Hence, the longitudinal variation of genera was similar between DS and AL. However, the genera exhibited distinct temporal changes between DS and AL within each microbiota type. Genera with increasing ICC values in AL indicated more stability in AL compared to in DS. The genera *Eubacterium*_g5 and *Sutterella* in M1, *Holdemanella* and *Coprococcu*s_g2 in M2, and *Frisingicoccus* in M3 were more stable in AL than those in DS. These genera changed in BASE, but they were stable in SHIP and RETURN. Therefore, these genera may be resistant to gut microbiota resilience in AL. Conversely, genera with decreasing ICC values in AL indicated greater variability in AL compared to DS. The genera UC_*Lachnospiraceae* (PAC001138_g) and *Bifidobacterium* in M1, *Barnesiella* and *Streptococcus* in M2, and *Butyricimonas* and *Bifidobacterium* in M3 changed more in AL than in DS. These variations in genera can result in disparate microbiota between HOME and RETURN in each microbiota type.

**FIGURE 5:**
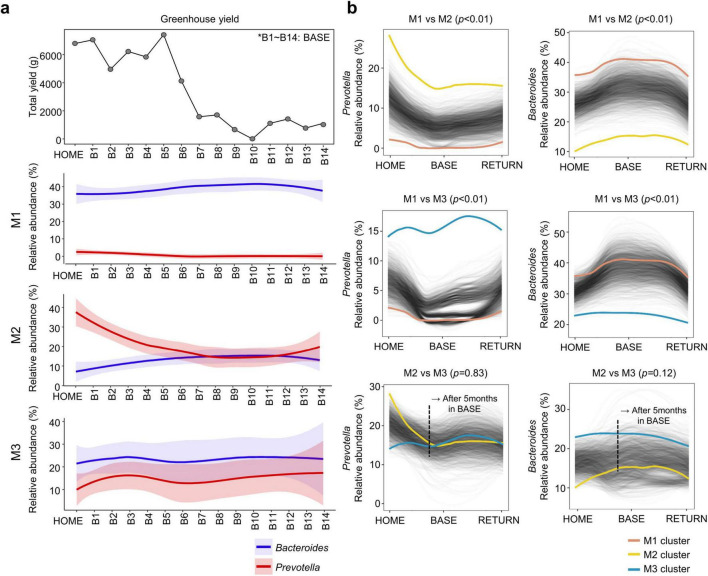
Correlation between fresh plant-based food sources at the Antarctic station and relative abundance of predominant genera (*Bacteroides* and *Prevotella*). **(a)** Longitudinal changes in total yield of plant cultivation in the greenhouse at the Antarctic station and relative abundance of *Bacteroides* and *Prevotella* in each microbiota type. **(b)** Longitudinal patterns of *Prevotella* and *Bacteroides* were compared between microbiota types. Differential abundance of *Prevotella* and *Bacteroides* was analyzed using the permutation-based package SpinectomeR. HOME, before participants departed from Korea; BASE, stay at Antarctic stations; RETURN, after return to Korea.

The decline in the relative abundance of *Prevotella* in M2 occurred at a similar time to the decline in vegetable production in the Antarctic station greenhouse (Figure 5a). However, the proportions of *Bacteroides* and *Prevotella* in M1 and M3 remained stable and were not correlated with the vegetable production decline. The difference in the relative abundance of *Prevotella* and *Bacteroides* between M2 and M3 reduced after 5 months in BASE (Figure 5b). Hence, the supply of a fresh plant-based diet influenced the *Prevotella*-dominant microbiota alone.

### 3.6 Longitudinal shifts in gut microbiota are mediated by highly variable genera within each microbiota type

To identify drivers for gut microbiota alterations, microbial interactions were analyzed using network analysis. Microbial interactions exhibited notable differences depending on microbiota types and sampling time points (Supplementary Figures S5–S7). Genera were divided into four groups based on the ICC values observed in DS and AL. Group A genera demonstrated consistent stability in both DS (ICC ≥ 0.5) and AL (ICC ≥ 0.5). In contrast, Group B genera exhibited stability in the DS (ICC ≥ 0.5), but variability in the AL (ICC < 0.5). Group C indicated variable genera in DS (ICC < 0.5) but stable genera in AL (ICC ≥ 0.5). Group D indicated variable genera in both DS (ICC < 0.5) and AL (ICC < 0.5). Complex interactions were identified in the M2 type, whereas interactions were relatively reduced in the M1 type in comparison to the other microbiota types. This indicated that the M1 type was relatively stable in DS and AL, with fewer microbial interactions.

Genus *Oscillibacter* in Group A, *Eubacterium*_g23 in Group B, and four genera (*Ruthenibacterium*, UC_*Lachnospiraceae*, *Roseburia*, and UC_*Peptostreptococcaceae*) in Group D were identified as keystones in the microbial interactions of M1 type in the DS (Supplementary Figure S5). *Longicatena* in Group C and three genera (*Escherichia*, *Pseudoflavonifractor*, and *Ruthenibacterium*) in Group D were identified as keystones in the microbial interaction of AL. *Bacteroides* in Group A and *Desulfovibrio* in Group D were identified as keystones in the interaction of M3 type in the DS (Supplementary Figure S7). PAC001048_g in Group C and *Ruminococcus* in Group D were identified as keystone taxa in the AL interaction. In contrast, no keystones (rank value > 0.3) were identified in interactions of the M2 type in both DS and AL (Supplementary Figure S6). Stable genera were identified as keystones in the microbial interactions of M1 and M3 types exclusively in the DS. In contrast, variable genera in Group D were identified as keystones in the microbial interactions in both DS and AL. In particular, the number of genera in Group D was greater than that of other groups in all longitudinal-shifted gut microbiota and interactions of the M2 type. These findings suggest that the observed differences in microbiota between the different microbiota types were driven by distinct microbial interactions within each microbiota. Longitudinal shifts in the gut microbiota could be mediated by relatively highly variable genera (ICC < 0.5 in both DS and AL).

### 3.7 Longitudinal variation in potential gut microbiota functions

**FIGURE 6:**
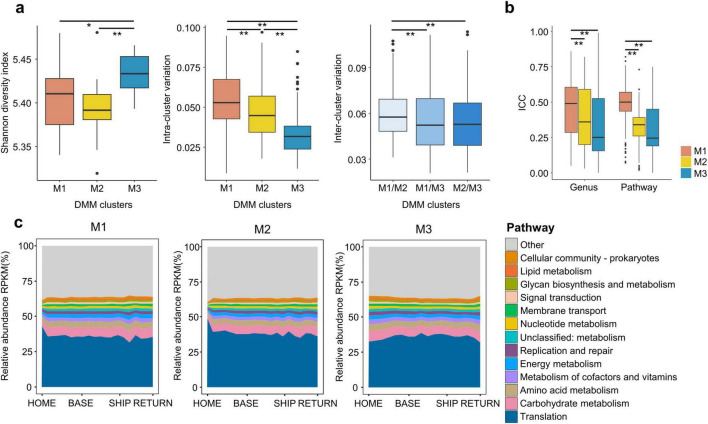
Comparing the gut microbiota functional potential among microbiota types. **(a)** Shannon diversity indices of predicted functional features before staying in the Antarctic bases were compared among microbiota types. Variations in predicted functional features before the stay within each microbiota type (intra-cluster variation) and between microbiota types (inter-cluster variation) were compared in box plots. **(b)** Intra-class correlation coefficients (ICCs) for genus and pathways were compared among microbiota types. **(c)** Longitudinal changes in overall pathway (1^st^ category of KEGG ortholog) based on predicted functional features were compared among microbiota types. Unclassified functional features in KEGG hierarchies were combined into the “others.” The *p*-values for two group comparisons were calculated using the Wilcoxon-rank sum test. HOME, before participants departed from Korea; BASE, stay at Antarctic stations; SHIP, ship voyage after leaving the stations; RETURN, after return to Korea; KEGG, Kyoto Encyclopedia of Genes and Genomes. *p < 0.05, **p < 0.01.

Temporal shifts in the gut microbiota differed based on the microbiota type before departure (HOME). Therefore, the longitudinal variation of functional potential was analyzed in each microbiota type and compared over time. The diversity of functional features was higher in the M3 type than in the other types, whereas the intra-cluster variation was higher in the M1 type (*p* < 0.05; Figure 6a). The inter-cluster variation was significantly higher between the M1 and M2 types than in any other pairwise comparison (*p* < 0.01). These findings were consistent with those observed in taxonomic features of HOME samples (Figure 1e). The disparity between inter-cluster variations in functional features was relatively small in comparison to that observed in taxonomic features. The ICC values exhibited a higher degree of consistency over time in the M1 type than in the other types, both in taxonomic and functional features (*p* < 0.01; Figure 6b). Although the variation values in functional features were comparable to those in taxonomic features, the relative abundances of potential functions in the overall pathway categories based on KEGG orthology exhibited a relatively stable pattern over time (Figure 6c).

## 4 Discussion

In this study, we analyzed the longitudinal alterations of gut microbiota from before departure to that after the participants stayed at Antarctic bases. We anticipated a reduction in gut microbiota diversity because of the limited availability of fresh food. However, the microbiota diversity increased during the participants’ stay, despite the existence of individual differences. This result is consistent with the increased diversity observed during Antarctic expeditions in previous studies (Moraes et al., 2023; Bhushan et al., 2019). The duration of stay at stations ranged from 2 to 16 months in this study, which indicates that limited fresh food supply in the station does not significantly influence gut microbiota diversity for approximately 1 year.

The gut microbiota of male participants was clustered into three distinct microbiota types at HOME, which were identified based on the dominant genera: *Bacteroides*-dominant, *Prevotella*-dominant, and *Bacteroides*/*Prevotella* co-dominant. Longitudinal gut microbiota changes differed among the microbiota types. The *Bacteroides*-dominant microbiota (M1 type) was relatively stable (ICC > 0.5), whereas the *Prevotell*a-dominant microbiota (M2 type) changed dynamically (ICC = 0.35) over time. The influence of exposure to extreme environments, including cold temperatures and limited food sources, on changes in the gut microbiota, specifically *Bacteroides* and *Prevotella*, has been reported in both animal and human studies (Karl et al., 2018a; Bai et al., 2022). *Bacteroides* and *Prevotella* are genera of the normal gut microbiota, and their abundance has been suggested to indicate a healthy gut microbiota (Hollister et al., 2014). An animal fat and protein-rich diet is associated with *Bacteroides* abundance, whereas a diet rich in carbohydrates and monosaccharides is associated with *Prevotella* abundance. Previous studies have reported a negative correlation between the relative abundance of *Bacteroides* and *Prevotella* (Moeller et al., 2014; Arumugam et al., 2011). These findings suggest that the gut microbiota at HOME may be influenced by dietary habits, and that gut microbiota changes at BASE may be influenced by environmental conditions of the Antarctic stations. Although the correlation between vegetable productions in the station greenhouse and changes in *Bacteroides* and *Prevotella* proportions differed among microbiota types, the variations and increased similarity between M2 and M3 were related to the total greenhouse yield. Consequently, the individual variability of gut microbiota in the DS and AL may be influenced by lifestyle, including diet.

Although *Prevotella* decreased at BASE because of dietary factors, shifts in the *Prevotella* proportion differed based on microbiota types. Specifically, the correlation between a decreased relative abundance of *Prevotella* and availability of fresh plant-based diets from the greenhouse was observed only in M2 (*Prevotella*-dominant microbiota). The ICC value of *Prevotella* was ≤ 0.44 in M2 but was ≥ 0.90 in M3 (*Bacteroides*/*Prevotella* co-dominant microbiota) in DS and AL. This indicates that microbiota shifts were not solely influenced by dietary factors but by various complex factors, including interactions between microbes in the gut. Therefore, gut microbiota alterations exhibited distinct patterns contingent upon microbiota composition. Ecological theory posits that diverse communities are more resilient and resistant to invasion by new species (Dill-McFarland et al., 2019; Levine and D’Antonio, 1999). However, our findings indicate that variation and resilience are not dependent on diversity but are influenced by microbiota composition. The genera that exhibited stability and variability differed among the microbiota types, and changes in these genera also differed between DS and AL. Our study also corroborates findings of a previous longitudinal study (Olsson et al., 2022) that dominant taxa are relatively stable, whereas rare taxa exhibit high variation. Although different genera exhibited ICCs > 0.75 and ICCs < 0.25 in each microbiota type, dominant genera exhibited high ICC values, whereas minor genera exhibited low ICC values in all microbiota types.

*Phascolarctobacterium* was consistently dominant and stable (ICC > 0.75) across all microbiota types. *Phascolarctobacterium* is a prevalent genus in the gut that produces propionate via utilizing succinate. Its beneficial effects have been reported in several studies (Wu et al., 2017; Li et al., 2022; Panasevich et al., 2016). *Phascolarctobacterium* has been associated with weight loss in an obesity treatment study (Muniz Pedrogo et al., 2018). Therefore, the absent correlation between BMI and gut microbiota alterations in this study could be attributed to *Phascolarctobacterium* stability in all participants. In contrast, *Escherichia* and *Streptococcus* were minor and highly variable genera (ICC < 0.25) in all microbiota types. The liberation of ecological niches in the gut can be reflected in the occasionally increasing rare taxa. However, fluctuations in these taxa may be a dynamic response to alterations in the gut environment, such as nutrient availability (Song et al., 2017). Previous studies have reported the susceptibility and fast response of Proteobacteria, including *Escherichia*, to environmental factors (Shin et al., 2015). *Streptococcus* is a conditionally rare taxon in various ecosystems (Lawson et al., 2015). The dominant genera, *Oscillibacter* in M1 and *Bacteroides* in M3, were identified as keystones in microbial interactions only in the DS. The highly variable minor genera were identified as keystones in both DS and AL. These findings suggest that microbiota alterations may be influenced by minor genera in response to changes in the gut environment over time. The degree of ICC values was higher in AL than that in the DS, indicating that the altered microbiota observed during the stay over 1 year did not rapidly restore the microbiota to its intrinsic state within an average of 1.4 months following the return to Korea.

Long-term stays in isolated Antarctic stations can cause cohabitation effects in gut microbiota among participants. Shared diets, proximity, and frequent physical contact have been proposed to be correlated with increasing microbiota similarity among participants during an expedition (Scott et al., 2013; Amato et al., 2017). Previous studies have indicated that genetically related individuals harbor more similar microbial communities than unrelated individuals, regardless of current cohabitation (Goodrich et al., 2014; Tims et al., 2013). Our results demonstrated gut microbiota conversion and its increasing similarity among participants during BASE compared to HOME. This discrepancy between previous studies and our study may be attributed to the isolated Antarctic stations where the study was conducted. However, the observed increase in gut microbiota similarities differed between microbiota types, with values ranging from 40 to 60% during their stay. The observed similarity values declined after leaving the stations, suggesting that the extremely isolated environment may facilitate cohabitation effects in gut microbiota among genetically unrelated participants over time.

The functional potential of the gut microbiota among healthy subjects is more conserved compared with the taxonomic composition (Turnbaugh et al., 2009). Although variations and ICC values of functional potential were similar to those of genus composition, the relative abundance of overall pathways was stable during and post-stay in this study, supporting that the functional redundancy of the gut microbiota is caused by the presence of essential pathways in several bacteria belonging to different taxa (Tian et al., 2020). Although the overall pathways were conserved in all microbiota types, subclass variations in pathways over time were also detected with low ICC values. The comparable outcomes of variation analysis between functional potential and taxonomic composition in this study may be attributed to the phylogenetic investigation of communities via reconstructing unobserved states (PICRUSt2) utilization. The functional potential of the gut microbiota is predicted based on 16S amplicon sequences, which are matched to reference genomes and most closely related genomes using a hidden state prediction algorithm in the PICRUSt2 (Douglas et al., 2020). Although PICRUSt2 utility has been demonstrated, the predicted functional potential should be interpreted with caution (Djemiel et al., 2022; Toole et al., 2021; Wang et al., 2022). Further studies using whole metagenome and metatranscriptome analyses are necessary to clarify the longitudinal functional changes in the gut microbiota while staying at the Antarctic stations.

Our findings should be interpretated within the broader context of isolated and confined environments (ICE), such as Antarctic research stations. Consistent with a recent systematic review (Klos et al., 2023), our study provides longitudinal insight into microbiome dynamics under ICE conditions, suggesting that microbiota composition may play a more critical role than overall diversity in determining microbial responses to environmental stressors. These findings underscore the importance of understanding gut microbial resilience in extreme settings and highlight the need for future studies that integrate microbiome data with physiological, psychological, and immunological parameters to better understand host adaptation in such environments.

This study has several limitations. First, clinical characteristics and individual habits (e.g., diet, sleep, and physical activity) were not collected because of the limited medical facilities available at the Antarctic stations and complex logistics of long-term stays. In particular, BMI data were available for only a subset of participants (16 out of 48), limiting our ability to assess the impact of body weight changes. As body weight fluctuations can influence gut microbiota, this remains a relevant limitation. Second, only male participants were included in this study. Third, this study focused on the bacteria in the gut microbiota. The influence of other microbes, such as archaea, viruses, and fungi, on the host-microbiome interactions must also be considered during long-term stays in the Antarctic bases. Furthermore, the integration of additional meta-omics techniques, such as metabolomics and metatranscriptomics, is necessary to elucidate the longitudinal alterations of the gut microbiome. Despite these limitations, our study identified that the intrinsic gut microbiota before the stay influenced shifts in microbiota during the stay at the Antarctic stations and after returning to Korea, as evidenced by the longitudinal study. Notably, the microbiota dominated by *Prevotella* is more susceptible to change than that dominated by *Bacteroides*. The nature of this study is exploratory and based on a limited number of participants due to the limited availability of members who travel to Antarctic stations. To the best of our knowledge, this study represents the longest investigation of gut microbiota alterations in a larger number of participants (48 males and 467 fecal samples), compared with previous studies (Jin et al., 2014; Cameron et al., 2023; Bhushan et al., 2019), and provides longitudinal gut microbiota dynamics of participants in an Antarctic research program over time.

## 5 Conclusion

In conclusion, long-term residence in Antarctic stations significantly influences the gut microbiota; variations are dependent on the intrinsic microbiota prior to staying in the stations. The isolated environment facilitated cohabitation effects among genetically unrelated individuals. Dominant taxa remained relatively stable, whereas minor taxa exhibited greater variability and mediated microbiota alterations. These findings can be utilized to predict the temporal dynamic of gut microbiota based on individuals’ microbiota. It can be used to maintain host health through the implementation of additional clinical translational studies. Although further meta-omics data and experimental validation are required, our findings highlight the significant impact of Antarctic station habitation on gut microbiota, enhancing our understanding of gut microbiota dynamics in response to extreme environmental changes.

## Data Availability

The original contributions presented in the study are publicly available. This data can be found here: https://www.ebi.ac.uk/ena, accession number PRJEB72775.
