# Supplementary material for: Longitudinal gut microbiota dynamics in Antarctic research mission crews
Source: Front Microbiol. 2025 May 22;16:1593617. doi: 10.3389/fmicb.2025.1593617 (PMC12142052; doi:10.3389/fmicb.2025.1593617)
Supplement: Supplementary file 1 [file Data_Sheet_1.docx]

Supplementary Material


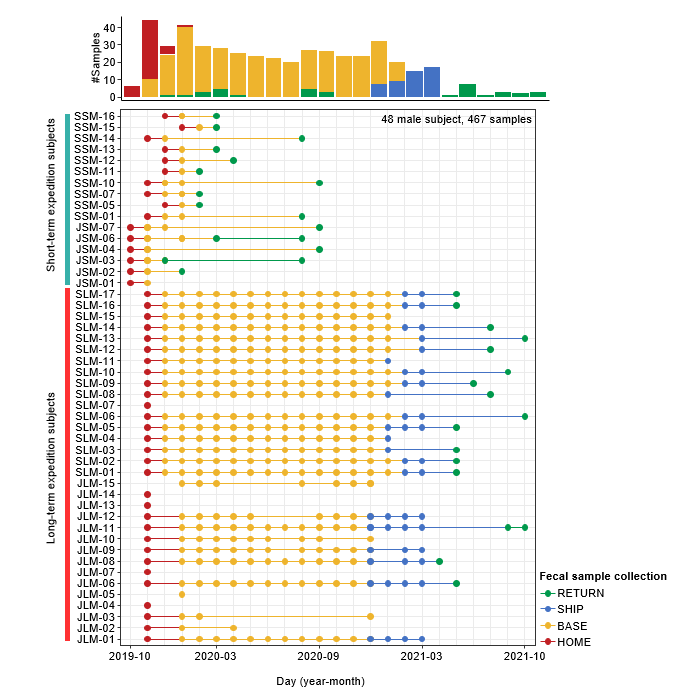


**Supplementary Figure S1. Overview of study subjects and longitudinal fecal sample collection schedule**. Fecal samples were collected before participants departed from Korea (HOME), during their stay at the Antarctic stations (BASE), during the ship voyage after leaving the stations (SHIP), and after returning to Korea (RETURN). A total of 467 fecal samples were collected from 48 participants who were Antarctic research program members.


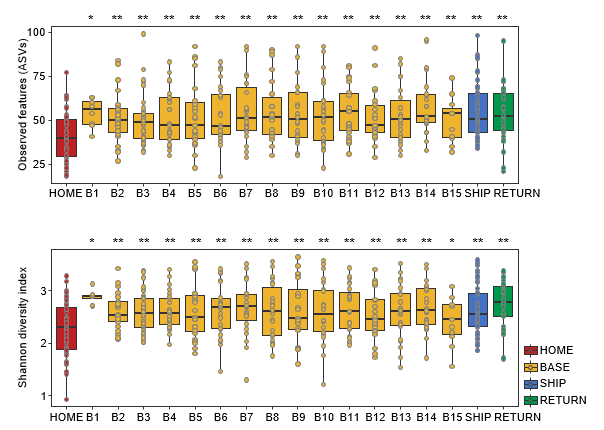


**Supplementary Figure S2.** **Longitudinal changes of observed ASVs and Shannon diversity indices in the gut microbiota.** Significant differences between the analyzed time point and HOME were calculated using the Wilcoxon-rank sum test. ASVs, amplicon sequence variants; HOME, before participants departed from Korea; BASE, stay at Antarctic stations; SHIP, ship voyage after leaving the stations; RETURN, after returning to Korea. **p* < 0.05, ***p* < 0.01.


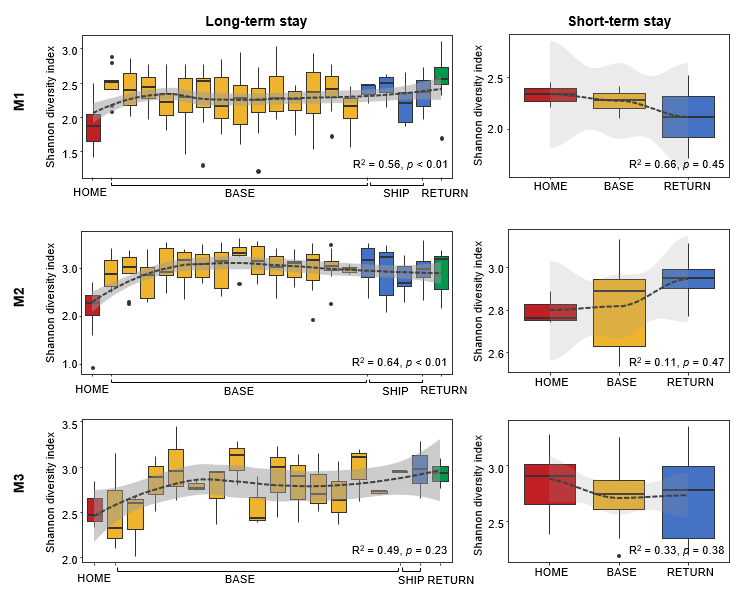


**Supplementary Figure S3.** **Longitudinal changes in gut microbiota diversity were analyzed for each microbiota type.** Participants who stayed at the stations for more than 13 months were classified as part of a long-term expedition, whereas those who stayed for less than three months were part of a short-term expedition. The slope of a log-linear model fitted to the diversity change over time represents the change rate. Gray line represents 95% confidence intervals (CIs) around the linear model. HOME, before participants departed from Korea; BASE, stay at Antarctic stations; SHIP, ship voyage after leaving the stations; RETURN, after returning to Korea.


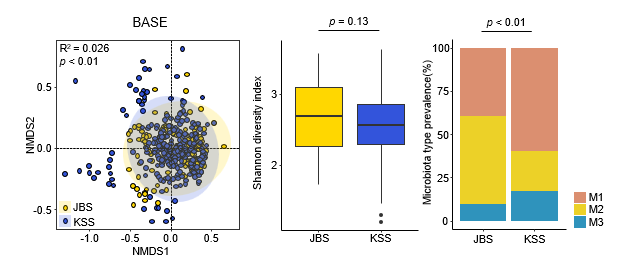


**Supplementary Figure S4.** **Comparing gut microbiota in male participants between two different stations (Jang Bogo and King Sejong) during the stay (BASE).** Compositional differences of the gut microbiota between two stations were analyzed in non-multidimensional scale (NMDS) plots. The *p*-value was calculated using permutational multivariate analysis of variance (PERMANOVA). Shannon diversity indices of the gut microbiota were compared between two stations in box plots. The composition of microbiota types was compared between two stations in bar plots. The microbiota type was determined by the microbiota before departure (HOME). The *p*-values for box and bar plots were calculated using the Wilcoxon-rank sum test. JBS, Jang Bogo Station; KSS, King Sejong Station; HOME, before participants departed from Korea; BASE, stay at Antarctic stations.


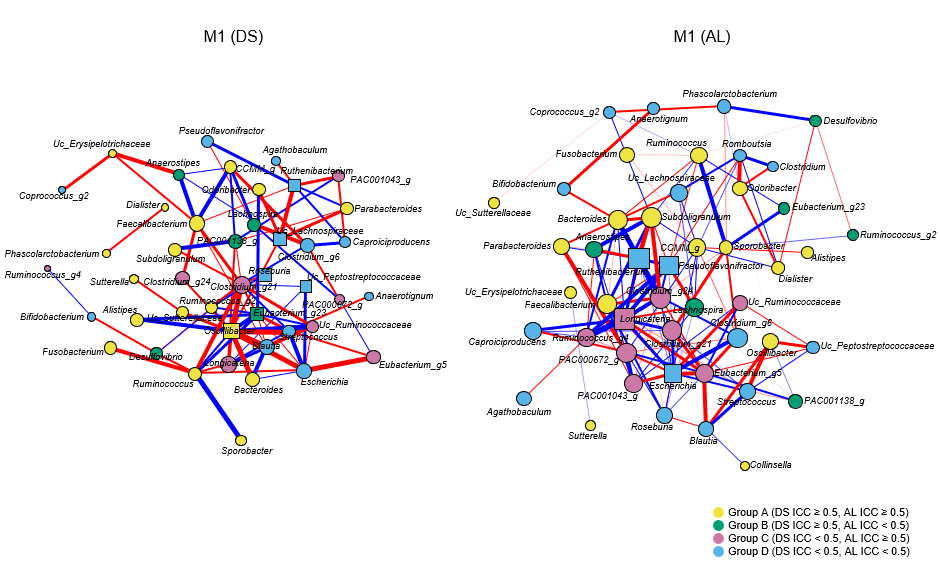


**Supplementary Figure S5.** **Network analysis of dominant genera in the gut microbiota of M1 type at DS and AL.** A total of 47 genera were selected by median abundance ≥ 0.1% and prevalence ≥ 30% in the M1 gut microbiota. Positive and negative correlations are indicated by blue and red edges, respectively. Edge thickness indicates FastSpar correlation, ranging from values of -0.5 to 0.5. Node sizes were scaled to the eigenvector centrality measure. Only significant correlations with *p* < 0.05 are shown. Network hubs identified by the PageRank algorithm with a rank value > 0.3 are marked with square symbols. Genera in Group A that exhibited ICCs ≥ 0.5 at both DS and AL are marked in yellow. Genera in Group B, which exhibited ICCs ≥ 0.5 at DS and ICCs < 0.5 at AL, are marked in green. Genera in Group C that had ICCs < 0.5 at DS and ICCs ≥ 0.5 at AL are marked in purple. Genera in Group D that had ICCs < 0.5 at both DS and AL are marked in light blue. DS, During Stay which included HOME and BASE samples; AL, After Leaving which included SHIP and RETURN samples; HOME, before participants departed from Korea; BASE, stay at Antarctic stations; SHIP, ship voyage after leaving the stations; RETURN, after returning to Korea; ICC, intra-class correlation coefficient.


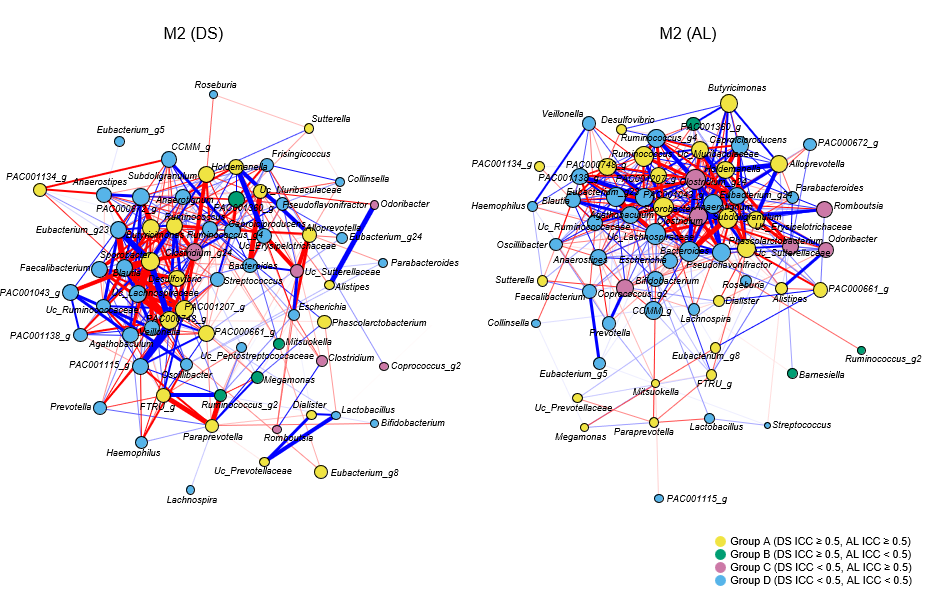


**Supplementary Figure S6.** **Network analysis of dominant genera in the M2 type gut microbiota at DS and AL.** A total of 65 genera were selected by median abundance ≥ 0.1% and prevalence ≥ 30% in the M2 gut microbiota. Positive and negative correlations are indicated by blue and red edges, respectively. Edge thickness indicates FastSpar correlation, ranging from values of -0.5 to 0.5. Node sizes were scaled to the eigenvector centrality measure. Only significant correlations with *p* < 0.05 are shown. No network hub was identified by the PageRank algorithm with a rank value > 0.3. Genera in Group A that exhibited ICCs ≥ 0.5 at both DS and AL are marked in yellow. Genera in Group B, which exhibited ICCs ≥ 0.5 at DS and ICCs < 0.5 at AL, are marked in green. Genera in Group C that had ICCs < 0.5 at DS and ICCs ≥ 0.5 at AL are marked in purple. Genera in Group D that had ICCs < 0.5 at both DS and AL are marked in light blue. DS, During Stay which included HOME and BASE samples; AL, After Leaving which included SHIP and RETURN samples; HOME, before participants departed from Korea; BASE, stay at Antarctic stations; SHIP, ship voyage after leaving the stations; RETURN, after returning to Korea; ICC, intra-class correlation coefficient.


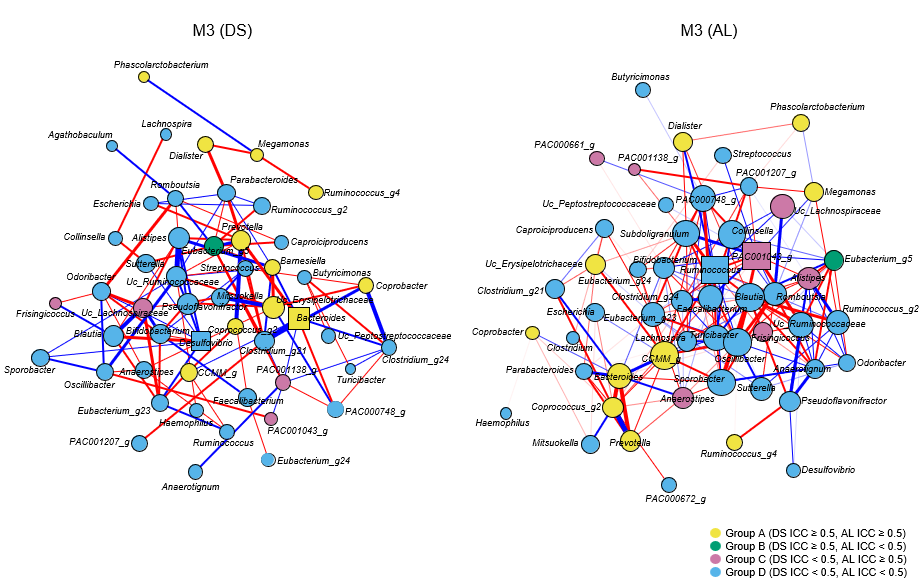


**Supplementary Figure S7.** **Network analysis of dominant genera in the M3 type gut microbiota at DS and AL.** A total of 55 genera were selected by median abundance ≥ 0.1% and prevalence ≥ 30% in the M3 gut microbiota. Positive and negative correlations are indicated by blue and red edges, respectively. Edge thickness indicates FastSpar correlation, ranging from values of -0.5 to 0.5. Node sizes were scaled to the eigenvector centrality measure. Only significant correlations with *p* < 0.05 are shown. Network hubs identified by the PageRank algorithm with a rank value > 0.3 are marked with square symbols. Genera in Group A that exhibited ICCs ≥ 0.5 at both DS and AL are marked in yellow. Genera in Group B, which exhibited ICCs ≥ 0.5 at DS and ICCs < 0.5 at AL, are marked in green. Genera in Group C that had ICCs < 0.5 at DS and ICCs ≥ 0.5 at AL are marked in purple. Genera in Group D that had ICCs < 0.5 at both DS and AL are marked in light blue. DS, During Stay which included HOME and BASE samples; AL, After Leaving which included SHIP and RETURN samples; HOME, before participants departed from Korea; BASE, stay at Antarctic stations; SHIP, ship voyage after leaving the stations; RETURN, after returning to Korea; ICC, intra-class correlation coefficient.

**Supplementary Table S1.** Inter- and intra-individual variances of dominant genera in male participants. Dominant genera were selected by median abundance ≥ 0.1% and prevalence ≥ 30% in all samples. UC, unclassified.

| Genus | *p*-value | | FDR | | coefficient  (fixed effect) | | variance in abundance, Total | Inter-individual variance (τ^2^) | Intra-individual variance (α^2^) | ICC |
| --- | --- | --- | --- | --- | --- | --- | --- | --- | --- | --- |
|  | Inter-individual | Intra-individual | Inter-individual | Intra-individual | Inter-individual | Intra-individual |  |  |  |  |
| *Megamonas* | 0.844 | 0.771 | 0.904 | 0.964 | 0.661 | -0.714 | 1.87 | 1.72 | 0.15 | 0.92 |
| *Prevotella* | 4.E-02 | 0.725 | 0.101 | 0.949 | 7.881 | 0.996 | 2.61 | 2.21 | 0.40 | 0.84 |
| *Phascolarctobacterium* | 0.823 | 0.664 | 0.904 | 0.913 | -0.592 | -0.841 | 1.25 | 1.04 | 0.21 | 0.83 |
| *Paraprevotella* | 0.833 | 0.068 | 0.904 | 0.382 | -0.513 | -3.154 | 1.08 | 0.86 | 0.22 | 0.80 |
| UC_Erysipelotrichaceae | 0.220 | 0.755 | 0.351 | 0.964 | 3.076 | -0.572 | 1.20 | 0.93 | 0.27 | 0.78 |
| *Alistipes* | 1.E-02 | 0.980 | 4.E-02 | 0.980 | -6.214 | 0.044 | 1.22 | 0.90 | 0.32 | 0.74 |
| *Dialister* | 0.855 | 0.113 | 0.904 | 0.444 | 0.448 | -2.750 | 1.16 | 0.84 | 0.32 | 0.72 |
| *Faecalibacterium* | 7.E-04 | 1.E-02 | 9.E-03 | 0.382 | -6.343 | -3.509 | 0.79 | 0.55 | 0.24 | 0.70 |
| *Bacteroides* | 1.E-06 | 3.E-02 | 7.E-05 | 0.382 | -4.190 | -1.417 | 0.19 | 0.13 | 0.06 | 0.68 |
| *Subdoligranulum* | 4.E-02 | 0.063 | 0.101 | 0.382 | -4.606 | -2.901 | 1.03 | 0.70 | 0.33 | 0.68 |
| *Butyricimonas* | 0.116 | 0.429 | 0.212 | 0.824 | -3.160 | -1.161 | 0.84 | 0.57 | 0.27 | 0.67 |
| *Sporobacter* | 0.229 | 0.492 | 0.351 | 0.872 | -2.730 | -1.120 | 1.06 | 0.71 | 0.35 | 0.67 |
| *Oscillibacter* | 2.E-03 | 0.055 | 1.E-02 | 0.382 | -6.528 | -2.647 | 0.99 | 0.63 | 0.36 | 0.64 |
| *Ruminococcus* | 5.E-02 | 0.212 | 0.116 | 0.686 | -4.967 | -2.255 | 1.38 | 0.88 | 0.50 | 0.64 |
| UC_Sutterellaceae | 0.689 | 0.239 | 0.863 | 0.730 | 0.849 | -1.795 | 0.95 | 0.61 | 0.34 | 0.64 |
| *Eubacterium*_g23 | 0.246 | 0.331 | 0.356 | 0.780 | -2.786 | -1.630 | 1.25 | 0.77 | 0.48 | 0.62 |
| *Collinsella* | 0.674 | 0.444 | 0.863 | 0.824 | -0.894 | 1.142 | 0.98 | 0.60 | 0.38 | 0.61 |
| UC_Ruminococcaceae (PAC000661_g) | 0.576 | 0.956 | 0.792 | 0.980 | -1.266 | -0.089 | 1.12 | 0.68 | 0.44 | 0.61 |
| UC_Erysipelotrichaceae (CCMM_g) | 9.E-04 | 0.578 | 1.E-02 | 0.898 | -7.339 | -0.949 | 1.23 | 0.74 | 0.49 | 0.60 |
| UC_Christensenellaceae (PAC001207_g) | 0.183 | 0.634 | 0.325 | 0.898 | -2.562 | -0.662 | 0.83 | 0.49 | 0.34 | 0.59 |
| *Clostridium*_g21 | 0.711 | 0.599 | 0.869 | 0.898 | 0.910 | 0.903 | 1.35 | 0.78 | 0.57 | 0.58 |
| *Sutterella* | 0.105 | 0.528 | 0.212 | 0.898 | -3.490 | -0.975 | 1.08 | 0.62 | 0.46 | 0.57 |
| *Parabacteroides* | 3.E-02 | 0.696 | 0.099 | 0.934 | -3.217 | -0.398 | 0.54 | 0.30 | 0.24 | 0.55 |
| *Ruminococcus*_g2 | 3.E-04 | 0.095 | 7.E-03 | 0.404 | -7.550 | -2.592 | 1.14 | 0.63 | 0.51 | 0.55 |
| *Longicatena* | 0.690 | 0.398 | 0.863 | 0.824 | 0.755 | 1.143 | 0.85 | 0.46 | 0.39 | 0.54 |
| *Odoribacter* | 1.E-02 | 0.301 | 4.E-02 | 0.780 | -4.768 | -1.368 | 0.92 | 0.45 | 0.47 | 0.49 |
| *Lactobacillus* | 3.E-02 | 0.836 | 0.099 | 0.968 | 3.807 | 0.268 | 0.82 | 0.40 | 0.42 | 0.49 |
| *Clostridium*_g24 | 0.797 | 0.813 | 0.904 | 0.968 | 0.533 | 0.333 | 1.09 | 0.52 | 0.57 | 0.48 |
| *Frisingicoccus* | 4.E-02 | 0.630 | 0.112 | 0.898 | -3.248 | -0.552 | 0.69 | 0.33 | 0.36 | 0.48 |
| *Ruminococcus*_g4 | 0.651 | 0.633 | 0.863 | 0.898 | 0.860 | -0.636 | 0.92 | 0.43 | 0.49 | 0.47 |
| *Desulfovibrio* | 0.078 | 0.073 | 0.179 | 0.382 | -3.080 | -2.163 | 0.81 | 0.38 | 0.43 | 0.47 |
| *Anaerostipes* | 1.E-02 | 0.904 | 4.E-02 | 0.975 | -3.817 | 0.132 | 0.67 | 0.29 | 0.38 | 0.44 |
| UC_Lachnospiraceae | 1.E-03 | 0.143 | 1.E-02 | 0.492 | -1.690 | -0.556 | 0.09 | 0.04 | 0.05 | 0.44 |
| UC_Ruminococcaceae | 4.E-02 | 0.277 | 0.101 | 0.780 | -3.194 | -1.215 | 0.66 | 0.29 | 0.37 | 0.44 |
| *Coprococcus*_g2 | 0.112 | 0.076 | 0.212 | 0.382 | -2.858 | -2.093 | 0.89 | 0.38 | 0.51 | 0.43 |
| *Lachnospira* | 5.E-03 | 4.E-02 | 3.E-02 | 0.382 | -4.692 | -2.212 | 0.85 | 0.35 | 0.50 | 0.41 |
| *Veillonella* | 4.E-02 | 0.637 | 0.101 | 0.898 | 3.057 | 0.501 | 0.66 | 0.27 | 0.39 | 0.41 |
| *Bifidobacterium* | 0.116 | 0.885 | 0.212 | 0.975 | -2.874 | -0.182 | 0.99 | 0.39 | 0.60 | 0.39 |
| UC_Lachnospiraceae (PAC001043_g) | 7.E-03 | 0.441 | 3.E-02 | 0.824 | -4.603 | -0.910 | 0.93 | 0.35 | 0.58 | 0.38 |
| *Eubacterium*_g5 | 5.E-03 | 2.E-02 | 3.E-02 | 0.382 | -4.489 | -2.476 | 0.86 | 0.30 | 0.56 | 0.35 |
| UC_Lachnospiraceae (PAC001138_g) | 0.104 | 0.054 | 0.212 | 0.382 | -2.669 | -2.147 | 0.93 | 0.30 | 0.63 | 0.32 |
| *Anaerotignum* | 3.E-03 | 0.060 | 2.E-02 | 0.382 | -3.996 | -1.760 | 0.71 | 0.20 | 0.51 | 0.28 |
| *Caproiciproducens* | 0.098 | 0.845 | 0.212 | 0.968 | -2.204 | -0.181 | 0.65 | 0.18 | 0.47 | 0.28 |
| UC_Ruminococcaceae (PAC000672_g) | 5.E-04 | 0.096 | 9.E-03 | 0.404 | -4.846 | -1.631 | 0.78 | 0.22 | 0.56 | 0.28 |
| *Ruthenibacterium* | 0.973 | 0.329 | 0.978 | 0.780 | 0.044 | 0.887 | 0.64 | 0.16 | 0.48 | 0.26 |
| *Haemophilus* | 0.774 | 0.449 | 0.904 | 0.824 | 0.442 | -0.776 | 0.86 | 0.22 | 0.64 | 0.26 |
| *Clostridium* | 0.324 | 0.965 | 0.457 | 0.980 | 1.672 | 0.050 | 1.08 | 0.27 | 0.81 | 0.25 |
| *Romboutsia* | 0.774 | 0.611 | 0.904 | 0.898 | -0.443 | 0.536 | 0.97 | 0.21 | 0.76 | 0.22 |
| *Streptococcus* | 0.215 | 0.138 | 0.351 | 0.492 | 1.675 | 1.366 | 0.79 | 0.17 | 0.62 | 0.21 |
| *Roseburia* | 8.E-03 | 0.313 | 4.E-02 | 0.780 | -4.029 | -1.055 | 0.97 | 0.21 | 0.76 | 0.21 |
| UC_Peptostreptococcaceae | 0.939 | 0.892 | 0.974 | 0.975 | 0.111 | -0.135 | 0.85 | 0.18 | 0.67 | 0.21 |
| *Escherichia* | 0.978 | 0.340 | 0.978 | 0.780 | 0.048 | 1.159 | 1.29 | 0.27 | 1.02 | 0.21 |
| *Blautia* | 0.240 | 0.797 | 0.356 | 0.968 | -0.551 | 0.082 | 0.10 | 0.02 | 0.08 | 0.20 |
| *Pseudoflavonifractor* | 0.189 | 0.930 | 0.325 | 0.980 | -1.525 | 0.066 | 0.56 | 0.11 | 0.45 | 0.20 |
| *Agathobaculum* | 0.230 | 0.356 | 0.351 | 0.783 | -1.322 | -0.678 | 0.68 | 0.09 | 0.59 | 0.13 |

**Supplementary Table S2.** Comparison of inter- and intra-individual variances of dominant genera in male participants across microbiota types. Dominant genera were selected by median abundance ≥ 0.1% and prevalence ≥ 30% in all male participants. Variances were calculated in each microbiota type. UC, unclassified.

| Genus | M1 microbiota type | | | | M2 microbiota type | | | | M3 microbiota type | | | |
| --- | --- | --- | --- | --- | --- | --- | --- | --- | --- | --- | --- | --- |
|  | variance in abundance, Total | Inter-individual variance (τ2) | Intra-individual variance (α2) | ICC | variance in abundance, Total | Inter-individual variance (τ2) | Intra-individual variance (α2) | ICC | variance in abundance, Total | Inter-individual variance (τ2) | Intra-individual variance (α2) | ICC |
| *Megamonas* | 1.00 | 0.98 | 0.02 | 0.98 | 1.74 | 1.31 | 0.43 | 0.75 | 2.33 | 2.31 | 0.02 | 0.99 |
| *Prevotella* | 1.09 | 0.64 | 0.45 | 0.59 | 0.60 | 0.21 | 0.39 | 0.35 | 2.69 | 2.41 | 0.28 | 0.90 |
| *Phascolarctobacterium* | 1.35 | 1.17 | 0.18 | 0.86 | 1.22 | 1.00 | 0.22 | 0.82 | 1.22 | 0.94 | 0.28 | 0.77 |
| *Paraprevotella* | 1.09 | 0.97 | 0.12 | 0.89 | 0.96 | 0.52 | 0.44 | 0.54 | 1.23 | 1.13 | 0.10 | 0.92 |
| UC_Erysipelotrichaceae | 0.59 | 0.36 | 0.23 | 0.61 | 1.40 | 1.10 | 0.30 | 0.79 | 1.46 | 1.15 | 0.31 | 0.79 |
| *Alistipes* | 1.40 | 1.16 | 0.24 | 0.83 | 1.09 | 0.71 | 0.38 | 0.65 | 0.55 | 0.10 | 0.45 | 0.19 |
| *Dialister* | 0.86 | 0.50 | 0.36 | 0.58 | 1.02 | 0.78 | 0.24 | 0.76 | 1.20 | 0.82 | 0.38 | 0.69 |
| *Faecalibacterium* | 1.51 | 1.13 | 0.38 | 0.75 | 0.11 | 0.01 | 0.10 | 0.05 | 0.06 | 0.01 | 0.05 | 0.15 |
| *Bacteroides* | 0.08 | 0.05 | 0.03 | 0.64 | 0.19 | 0.06 | 0.13 | 0.31 | 0.15 | 0.10 | 0.05 | 0.67 |
| *Subdoligranulum* | 1.28 | 0.85 | 0.43 | 0.66 | 0.66 | 0.39 | 0.27 | 0.59 | 0.17 | 0.01 | 0.16 | 0.06 |
| *Butyricimonas* | 0.49 | 0.29 | 0.20 | 0.59 | 1.07 | 0.76 | 0.31 | 0.71 | 0.92 | 0.48 | 0.44 | 0.52 |
| *Sporobacter* | 0.62 | 0.36 | 0.26 | 0.57 | 1.17 | 0.70 | 0.47 | 0.60 | 0.60 | 0.18 | 0.42 | 0.30 |
| *Oscillibacter* | 1.14 | 0.77 | 0.37 | 0.67 | 0.63 | 0.22 | 0.41 | 0.35 | 0.28 | 0.01 | 0.27 | 0.02 |
| *Ruminococcus* | 1.08 | 0.60 | 0.48 | 0.56 | 1.05 | 0.55 | 0.50 | 0.52 | 0.79 | 0.20 | 0.59 | 0.25 |
| UC_Sutterellaceae | 1.22 | 0.97 | 0.25 | 0.80 | 0.93 | 0.38 | 0.55 | 0.41 | 0.32 | 0.10 | 0.22 | 0.32 |
| *Eubacterium*_g23 | 1.11 | 0.64 | 0.47 | 0.58 | 0.93 | 0.41 | 0.52 | 0.44 | 0.67 | 0.23 | 0.44 | 0.34 |
| *Collinsella* | 1.10 | 0.82 | 0.28 | 0.75 | 0.85 | 0.29 | 0.56 | 0.34 | 0.61 | 0.23 | 0.38 | 0.38 |
| UC_Ruminococcaceae (PAC000661_g) | 0.62 | 0.22 | 0.40 | 0.36 | 1.34 | 0.94 | 0.40 | 0.70 | 1.42 | 0.77 | 0.65 | 0.54 |
| UC_Erysipelotrichaceae (CCMM_g) | 1.54 | 1.06 | 0.48 | 0.69 | 0.92 | 0.32 | 0.60 | 0.35 | 0.89 | 0.59 | 0.30 | 0.66 |
| UC_Christensenellaceae (PAC001207_g) | 0.32 | 0.14 | 0.18 | 0.44 | 1.03 | 0.54 | 0.49 | 0.52 | 0.68 | 0.12 | 0.56 | 0.18 |
| *Clostridium*_g21 | 1.01 | 0.37 | 0.64 | 0.36 | 0.78 | 0.32 | 0.46 | 0.41 | 0.86 | 0.29 | 0.57 | 0.34 |
| *Sutterella* | 1.49 | 0.89 | 0.60 | 0.60 | 0.76 | 0.54 | 0.22 | 0.71 | 0.51 | 0.04 | 0.47 | 0.07 |
| *Parabacteroides* | 0.97 | 0.71 | 0.26 | 0.73 | 0.34 | 0.06 | 0.28 | 0.18 | 0.14 | 0.05 | 0.09 | 0.33 |
| *Ruminococcus*_g2 | 1.19 | 0.61 | 0.58 | 0.51 | 0.87 | 0.46 | 0.41 | 0.53 | 0.95 | 0.44 | 0.51 | 0.46 |
| *Longicatena* | 0.77 | 0.33 | 0.44 | 0.43 | 0.56 | 0.15 | 0.41 | 0.27 | 0.27 | 0.07 | 0.20 | 0.26 |
| *Odoribacter* | 0.94 | 0.53 | 0.41 | 0.56 | 0.82 | 0.31 | 0.51 | 0.38 | 0.58 | 0.00 | 0.58 | 0.00 |
| *Lactobacillus* | 0.97 | 0.64 | 0.33 | 0.66 | 0.95 | 0.38 | 0.57 | 0.40 | 0.41 | 0.01 | 0.40 | 0.03 |
| *Clostridium*_g24 | 0.87 | 0.37 | 0.50 | 0.43 | 0.91 | 0.29 | 0.62 | 0.32 | 0.87 | 0.18 | 0.69 | 0.20 |
| *Frisingicoccus* | 0.57 | 0.25 | 0.32 | 0.44 | 0.55 | 0.16 | 0.39 | 0.30 | 0.53 | 0.12 | 0.41 | 0.22 |
| *Ruminococcus*_g4 | 1.17 | 0.57 | 0.60 | 0.49 | 0.47 | 0.02 | 0.45 | 0.05 | 1.05 | 0.84 | 0.21 | 0.80 |
| *Desulfovibrio* | 0.96 | 0.52 | 0.44 | 0.54 | 0.87 | 0.47 | 0.40 | 0.54 | 0.62 | 0.15 | 0.47 | 0.24 |
| *Anaerostipes* | 0.57 | 0.28 | 0.29 | 0.49 | 0.88 | 0.26 | 0.62 | 0.29 | 0.41 | 0.21 | 0.20 | 0.51 |
| UC_Lachnospiraceae | 0.12 | 0.06 | 0.06 | 0.51 | 0.05 | 0.01 | 0.04 | 0.12 | 0.02 | 0.00 | 0.02 | 0.10 |
| UC_Ruminococcaceae | 0.70 | 0.34 | 0.36 | 0.48 | 0.64 | 0.24 | 0.40 | 0.38 | 0.44 | 0.09 | 0.35 | 0.20 |
| *Coprococcus*_g2 | 1.05 | 0.45 | 0.60 | 0.43 | 0.63 | 0.22 | 0.41 | 0.36 | 0.87 | 0.46 | 0.41 | 0.53 |
| *Lachnospira* | 1.25 | 0.62 | 0.63 | 0.50 | 0.59 | 0.11 | 0.48 | 0.19 | 0.15 | 0.02 | 0.13 | 0.15 |
| *Veillonella* | 0.70 | 0.39 | 0.31 | 0.56 | 0.55 | 0.11 | 0.44 | 0.20 | 0.75 | 0.22 | 0.53 | 0.30 |
| *Bifidobacterium* | 1.02 | 0.42 | 0.60 | 0.41 | 0.98 | 0.25 | 0.73 | 0.26 | 0.44 | 0.08 | 0.36 | 0.18 |
| UC_Lachnospiraceae (PAC001043_g) | 1.26 | 0.59 | 0.67 | 0.47 | 0.71 | 0.26 | 0.45 | 0.36 | 0.66 | 0.09 | 0.57 | 0.13 |
| *Eubacterium*_g5 | 1.12 | 0.50 | 0.62 | 0.45 | 0.66 | 0.02 | 0.64 | 0.03 | 0.60 | 0.39 | 0.21 | 0.64 |
| UC_Lachnospiraceae (PAC001138_g) | 1.11 | 0.57 | 0.54 | 0.51 | 0.76 | 0.02 | 0.74 | 0.03 | 0.95 | 0.25 | 0.70 | 0.26 |
| *Anaerotignum* | 0.84 | 0.30 | 0.54 | 0.36 | 0.60 | 0.11 | 0.49 | 0.18 | 0.60 | 0.10 | 0.50 | 0.17 |
| *Caproiciproducens* | 0.51 | 0.02 | 0.49 | 0.05 | 0.72 | 0.29 | 0.43 | 0.41 | 0.65 | 0.14 | 0.51 | 0.22 |
| UC_Ruminococcaceae (PAC000672_g) | 0.82 | 0.22 | 0.60 | 0.27 | 0.80 | 0.26 | 0.54 | 0.33 | 0.60 | 0.16 | 0.44 | 0.27 |
| *Ruthenibacterium* | 0.79 | 0.24 | 0.55 | 0.30 | 0.52 | 0.07 | 0.45 | 0.14 | 0.43 | 0.12 | 0.31 | 0.27 |
| *Haemophilus* | 0.65 | 0.15 | 0.50 | 0.22 | 0.89 | 0.14 | 0.75 | 0.16 | 0.97 | 0.16 | 0.81 | 0.16 |
| *Clostridium* | 1.08 | 0.27 | 0.81 | 0.25 | 1.15 | 0.39 | 0.76 | 0.34 | 0.87 | 0.00 | 0.87 | 0.00 |
| *Romboutsia* | 1.12 | 0.30 | 0.82 | 0.26 | 0.75 | 0.09 | 0.66 | 0.12 | 0.90 | 0.15 | 0.75 | 0.17 |
| *Streptococcus* | 0.79 | 0.17 | 0.62 | 0.22 | 0.77 | 0.16 | 0.61 | 0.20 | 0.74 | 0.11 | 0.63 | 0.15 |
| *Roseburia* | 1.31 | 0.34 | 0.97 | 0.26 | 0.66 | 0.06 | 0.60 | 0.09 | 0.48 | 0.04 | 0.44 | 0.09 |
| UC_Peptostreptococcaceae | 0.89 | 0.18 | 0.71 | 0.20 | 0.79 | 0.13 | 0.66 | 0.17 | 0.95 | 0.38 | 0.57 | 0.40 |
| *Escherichia* | 1.45 | 0.34 | 1.11 | 0.23 | 1.04 | 0.17 | 0.87 | 0.16 | 1.08 | 0.08 | 1.00 | 0.08 |
| *Blautia* | 0.12 | 0.03 | 0.09 | 0.25 | 0.09 | 0.01 | 0.08 | 0.11 | 0.05 | 0.01 | 0.04 | 0.17 |
| *Pseudoflavonifractor* | 0.68 | 0.18 | 0.50 | 0.26 | 0.44 | 0.04 | 0.40 | 0.10 | 0.49 | 0.07 | 0.42 | 0.15 |
| *Agathobaculum* | 0.70 | 0.15 | 0.55 | 0.21 | 0.69 | 0.02 | 0.67 | 0.03 | 0.65 | 0.05 | 0.60 | 0.07 |
| UC_Ruminococcaceae (FTRU_g) | 0.64 | 0.38 | 0.26 | 0.60 | 0.70 | 0.40 | 0.30 | 0.57 | 0.36 | 0.05 | 0.31 | 0.14 |

**Supplementary Table S3.** Inter- and intra-individual variances of dominant genera in the M1 gut microbiota across time points. Dominant genera were selected by median abundance ≥ 0.1% and prevalence ≥ 30% in the M1 type gut microbiota. Variances were calculated at each time point. UC, unclassified; DS, During Stay which included HOME and BASE samples; AL, After Leaving which included SHIP and RETURN samples; HOME, before participants departed from Korea; BASE, stay at Antarctic stations; SHIP, ship voyage after leaving the stations; RETURN, after returning to Korea; ICC, intra-class correlation coefficient.

| Genus | All period | | | ICC | DS | | | ICC | AL | | | ICC |
| --- | --- | --- | --- | --- | --- | --- | --- | --- | --- | --- | --- | --- |
|  | variance in abundance, Total | Inter-individual variance (τ2) | Intra-individual variance (α2) |  | variance in abundance, Total | Inter-individual variance (τ2) | Intra-individual variance (α2) |  | variance in abundance, Total | Inter-individual variance (τ2) | Intra-individual variance (α2) |  |
| *Phascolarctobacterium* | 2.21 | 1.17 | 0.18 | 0.86 | 1.33 | 1.16 | 0.17 | 0.87 | 1.54 | 1.37 | 0.17 | 0.89 |
| *Alistipes* | 2.23 | 1.16 | 0.24 | 0.83 | 1.35 | 1.12 | 0.23 | 0.83 | 1.36 | 1.15 | 0.21 | 0.84 |
| UC_Sutterellaceae | 2.02 | 0.97 | 0.25 | 0.80 | 1.21 | 0.97 | 0.24 | 0.80 | 1.34 | 1.05 | 0.29 | 0.79 |
| *Collinsella* | 1.85 | 0.82 | 0.28 | 0.75 | 1.07 | 0.79 | 0.28 | 0.74 | 1.13 | 0.90 | 0.23 | 0.80 |
| *Faecalibacterium* | 2.26 | 1.13 | 0.38 | 0.75 | 1.47 | 1.06 | 0.41 | 0.72 | 1.74 | 1.41 | 0.33 | 0.81 |
| *Parabacteroides* | 1.70 | 0.71 | 0.26 | 0.73 | 0.88 | 0.65 | 0.23 | 0.74 | 0.93 | 0.50 | 0.43 | 0.53 |
| UC_Erysipelotrichaceae (CCMM_g) | 2.23 | 1.06 | 0.48 | 0.69 | 1.57 | 1.09 | 0.48 | 0.69 | 1.61 | 1.33 | 0.28 | 0.83 |
| *Oscillibacter* | 1.81 | 0.77 | 0.37 | 0.67 | 1.11 | 0.73 | 0.38 | 0.65 | 1.11 | 0.78 | 0.33 | 0.70 |
| *Fusobacterium* | 2.01 | 0.90 | 0.44 | 0.67 | 1.37 | 0.99 | 0.38 | 0.72 | 1.74 | 1.04 | 0.70 | 0.60 |
| *Subdoligranulum* | 1.94 | 0.85 | 0.43 | 0.66 | 1.27 | 0.82 | 0.45 | 0.65 | 1.22 | 0.96 | 0.26 | 0.79 |
| *Bacteroides* | 0.72 | 0.05 | 0.03 | 0.64 | 0.07 | 0.04 | 0.03 | 0.62 | 0.09 | 0.05 | 0.04 | 0.57 |
| UC_Erysipelotrichaceae | 1.20 | 0.36 | 0.23 | 0.61 | 0.58 | 0.39 | 0.19 | 0.67 | 0.62 | 0.37 | 0.25 | 0.59 |
| *Sutterella* | 2.09 | 0.89 | 0.60 | 0.60 | 1.49 | 0.86 | 0.63 | 0.58 | 1.72 | 1.37 | 0.35 | 0.80 |
| *Eubacterium*_g23 | 1.69 | 0.64 | 0.47 | 0.58 | 1.10 | 0.66 | 0.44 | 0.60 | 1.23 | 0.53 | 0.70 | 0.43 |
| *Dialister* | 1.44 | 0.50 | 0.36 | 0.58 | 0.84 | 0.47 | 0.37 | 0.56 | 0.91 | 0.58 | 0.33 | 0.64 |
| *Sporobacter* | 1.19 | 0.36 | 0.26 | 0.57 | 0.62 | 0.33 | 0.29 | 0.54 | 0.58 | 0.29 | 0.29 | 0.50 |
| *Odoribacter* | 1.50 | 0.53 | 0.41 | 0.56 | 0.93 | 0.52 | 0.41 | 0.56 | 0.99 | 0.51 | 0.48 | 0.52 |
| *Ruminococcus* | 1.64 | 0.60 | 0.48 | 0.56 | 1.08 | 0.58 | 0.50 | 0.54 | 0.93 | 0.51 | 0.42 | 0.55 |
| *Desulfovibrio* | 1.50 | 0.52 | 0.44 | 0.54 | 0.97 | 0.56 | 0.41 | 0.58 | 1.00 | 0.45 | 0.55 | 0.45 |
| UC_Lachnospiraceae (PAC001138_g) | 1.62 | 0.57 | 0.54 | 0.51 | 1.20 | 0.72 | 0.48 | 0.60 | 0.90 | 0.29 | 0.61 | 0.32 |
| UC_Lachnospiraceae | 0.63 | 0.06 | 0.06 | 0.51 | 0.12 | 0.06 | 0.06 | 0.48 | 0.12 | 0.05 | 0.07 | 0.41 |
| *Ruminococcus*_g2 | 1.70 | 0.61 | 0.58 | 0.51 | 1.19 | 0.62 | 0.57 | 0.52 | 1.16 | 0.55 | 0.61 | 0.48 |
| *Lachnospira* | 1.75 | 0.62 | 0.63 | 0.50 | 1.17 | 0.58 | 0.59 | 0.50 | 1.38 | 0.56 | 0.82 | 0.40 |
| *Anaerostipes* | 1.06 | 0.28 | 0.29 | 0.49 | 0.55 | 0.31 | 0.24 | 0.57 | 0.58 | 0.25 | 0.33 | 0.42 |
| *Ruminococcus*_g4 | 1.66 | 0.57 | 0.60 | 0.49 | 1.16 | 0.57 | 0.59 | 0.49 | 1.05 | 0.73 | 0.32 | 0.70 |
| UC_Ruminococcaceae | 1.18 | 0.34 | 0.36 | 0.48 | 0.69 | 0.32 | 0.37 | 0.47 | 0.74 | 0.43 | 0.31 | 0.58 |
| UC_Lachnospiraceae (PAC001043_g) | 1.73 | 0.59 | 0.67 | 0.47 | 1.27 | 0.56 | 0.71 | 0.44 | 1.12 | 0.73 | 0.39 | 0.65 |
| *Eubacterium*_g5 | 1.57 | 0.50 | 0.62 | 0.45 | 1.05 | 0.38 | 0.67 | 0.36 | 1.02 | 0.85 | 0.17 | 0.83 |
| *Clostridium*_g24 | 1.30 | 0.37 | 0.50 | 0.43 | 0.87 | 0.38 | 0.49 | 0.44 | 1.06 | 0.60 | 0.46 | 0.56 |
| *Coprococcus*_g2 | 1.48 | 0.45 | 0.60 | 0.43 | 1.05 | 0.42 | 0.63 | 0.40 | 0.93 | 0.43 | 0.50 | 0.46 |
| *Longicatena* | 1.20 | 0.33 | 0.44 | 0.43 | 0.80 | 0.37 | 0.43 | 0.46 | 0.64 | 0.41 | 0.23 | 0.65 |
| *Bifidobacterium* | 1.43 | 0.42 | 0.60 | 0.41 | 1.02 | 0.49 | 0.53 | 0.48 | 0.92 | 0.20 | 0.72 | 0.22 |
| *Anaerotignum* | 1.20 | 0.30 | 0.54 | 0.36 | 0.86 | 0.31 | 0.55 | 0.36 | 0.90 | 0.12 | 0.78 | 0.13 |
| *Clostridium*_g21 | 1.37 | 0.37 | 0.64 | 0.36 | 1.04 | 0.35 | 0.69 | 0.33 | 0.76 | 0.42 | 0.34 | 0.55 |
| *Ruthenibacterium* | 1.09 | 0.24 | 0.55 | 0.30 | 0.76 | 0.24 | 0.52 | 0.32 | 0.86 | 0.32 | 0.54 | 0.37 |
| UC_Ruminococcaceae (PAC000672_g) | 1.09 | 0.22 | 0.60 | 0.27 | 0.80 | 0.20 | 0.60 | 0.25 | 0.99 | 0.52 | 0.47 | 0.52 |
| *Roseburia* | 1.57 | 0.34 | 0.97 | 0.26 | 1.27 | 0.40 | 0.87 | 0.32 | 1.48 | 0.37 | 1.11 | 0.25 |
| *Romboutsia* | 1.38 | 0.30 | 0.82 | 0.26 | 1.09 | 0.34 | 0.75 | 0.31 | 1.32 | 0.40 | 0.92 | 0.30 |
| *Pseudoflavonifractor* | 0.94 | 0.18 | 0.50 | 0.26 | 0.72 | 0.20 | 0.52 | 0.27 | 0.40 | 0.14 | 0.26 | 0.34 |
| *Clostridium* | 1.33 | 0.27 | 0.81 | 0.25 | 1.10 | 0.29 | 0.81 | 0.26 | 1.01 | 0.22 | 0.79 | 0.22 |
| *Blautia* | 0.37 | 0.03 | 0.09 | 0.25 | 0.10 | 0.02 | 0.08 | 0.21 | 0.13 | 0.04 | 0.09 | 0.32 |
| *Escherichia* | 1.68 | 0.34 | 1.11 | 0.23 | 1.51 | 0.42 | 1.09 | 0.28 | 1.41 | 0.16 | 1.25 | 0.11 |
| *Streptococcus* | 1.01 | 0.17 | 0.62 | 0.22 | 0.77 | 0.22 | 0.55 | 0.29 | 0.90 | 0.12 | 0.78 | 0.13 |
| *Agathobaculum* | 0.91 | 0.15 | 0.55 | 0.21 | 0.68 | 0.15 | 0.53 | 0.23 | 0.64 | 0.11 | 0.53 | 0.18 |
| UC_Peptostreptococcaceae | 1.09 | 0.18 | 0.71 | 0.20 | 0.87 | 0.20 | 0.67 | 0.23 | 0.95 | 0.00 | 0.95 | 0.00 |
| *Clostridium*_g6 | 0.86 | 0.12 | 0.55 | 0.19 | 0.64 | 0.13 | 0.51 | 0.20 | 0.83 | 0.35 | 0.48 | 0.42 |
| *Caproiciproducens* | 0.56 | 0.02 | 0.49 | 0.05 | 0.51 | 0.02 | 0.49 | 0.04 | 0.44 | 0.02 | 0.42 | 0.04 |

**Supplementary Table S4.** Inter- and intra-individual variances of dominant genera in the M2 type gut microbiota across time points. Dominant genera were selected by median abundance ≥ 0.1% and prevalence ≥ 30% in the M2 gut microbiota. Variances were calculated at each time point. UC, unclassified; DS, During Stay which included HOME and BASE samples; AL, After Leaving which included SHIP and RETURN samples; HOME, before participants departed from Korea; BASE, stay at the Antarctic stations; SHIP, ship voyage after leaving the stations; RETURN, after returning to Korea; ICC, intra-class correlation coefficient.

| Genus | All period | | | ICC | DS | | | ICC | AL | | | ICC |
| --- | --- | --- | --- | --- | --- | --- | --- | --- | --- | --- | --- | --- |
|  | variance in abundance, Total | Inter-individual variance (τ2) | Intra-individual variance (α2) |  | variance in abundance, Total | Inter-individual variance (τ2) | Intra-individual variance (α2) |  | variance in abundance, Total | Inter-individual variance (τ2) | Intra-individual variance (α2) |  |
| *Phascolarctobacterium* | 1.22 | 1.00 | 0.22 | 0.82 | 1.28 | 1.07 | 0.21 | 0.84 | 1.26 | 1.08 | 0.18 | 0.86 |
| *Alloprevotella* | 1.64 | 1.31 | 0.33 | 0.80 | 1.59 | 1.38 | 0.21 | 0.87 | 1.75 | 1.35 | 0.40 | 0.77 |
| UC_Muribaculaceae | 1.27 | 1.00 | 0.27 | 0.79 | 1.26 | 1.05 | 0.21 | 0.84 | 1.33 | 0.89 | 0.44 | 0.67 |
| UC_Erysipelotrichaceae | 1.40 | 1.10 | 0.30 | 0.79 | 1.34 | 1.14 | 0.20 | 0.85 | 1.53 | 1.12 | 0.41 | 0.73 |
| *Dialister* | 1.02 | 0.78 | 0.24 | 0.76 | 1.01 | 0.78 | 0.23 | 0.77 | 1.19 | 0.99 | 0.20 | 0.83 |
| *Megamonas* | 1.74 | 1.31 | 0.43 | 0.75 | 1.65 | 1.32 | 0.33 | 0.80 | 1.94 | 1.46 | 0.48 | 0.75 |
| *Butyricimonas* | 1.07 | 0.76 | 0.31 | 0.71 | 1.04 | 0.76 | 0.28 | 0.73 | 1.03 | 0.65 | 0.38 | 0.63 |
| *Sutterella* | 0.76 | 0.54 | 0.22 | 0.71 | 0.72 | 0.54 | 0.18 | 0.75 | 1.12 | 0.72 | 0.40 | 0.65 |
| UC_Bacteroidales (PAC001134_g) | 0.85 | 0.60 | 0.25 | 0.70 | 0.84 | 0.61 | 0.23 | 0.73 | 0.98 | 0.69 | 0.29 | 0.70 |
| UC_Ruminococcaceae (PAC000661_g) | 1.34 | 0.94 | 0.40 | 0.70 | 1.35 | 0.91 | 0.44 | 0.67 | 1.58 | 1.43 | 0.15 | 0.91 |
| UC_Prevotellaceae | 0.74 | 0.50 | 0.24 | 0.68 | 0.75 | 0.50 | 0.25 | 0.67 | 0.79 | 0.56 | 0.23 | 0.71 |
| *Mitsuokella* | 0.73 | 0.49 | 0.24 | 0.68 | 0.72 | 0.57 | 0.15 | 0.80 | 0.79 | 0.43 | 0.36 | 0.55 |
| *Alistipes* | 1.09 | 0.71 | 0.38 | 0.65 | 1.07 | 0.73 | 0.34 | 0.68 | 1.09 | 0.73 | 0.36 | 0.67 |
| *Holdemanella* | 1.17 | 0.75 | 0.42 | 0.64 | 1.10 | 0.68 | 0.42 | 0.62 | 1.29 | 1.15 | 0.14 | 0.89 |
| *Barnesiella* | 0.76 | 0.47 | 0.29 | 0.62 | 0.74 | 0.49 | 0.25 | 0.66 | 0.49 | 0.13 | 0.36 | 0.27 |
| *Sporobacter* | 1.17 | 0.70 | 0.47 | 0.60 | 1.12 | 0.67 | 0.45 | 0.60 | 1.33 | 0.90 | 0.43 | 0.68 |
| *Subdoligranulum* | 0.66 | 0.39 | 0.27 | 0.59 | 0.58 | 0.32 | 0.26 | 0.55 | 0.95 | 0.75 | 0.20 | 0.79 |
| UC_Ruminococcaceae (FTRU_g) | 0.70 | 0.40 | 0.30 | 0.57 | 0.70 | 0.41 | 0.29 | 0.58 | 0.76 | 0.41 | 0.35 | 0.54 |
| UC_Sutterellaceae | 0.79 | 0.38 | 0.41 | 0.55 | 0.99 | 0.41 | 0.58 | 0.42 | 0.77 | 0.40 | 0.37 | 0.52 |
| *Paraprevotella* | 0.96 | 0.52 | 0.44 | 0.54 | 0.93 | 0.54 | 0.39 | 0.58 | 1.05 | 0.60 | 0.45 | 0.57 |
| UC_Ruminococcaceae (PAC000748_g) | 0.94 | 0.51 | 0.43 | 0.54 | 0.93 | 0.51 | 0.42 | 0.55 | 0.90 | 0.55 | 0.35 | 0.61 |
| *Desulfovibrio* | 0.87 | 0.47 | 0.40 | 0.54 | 0.87 | 0.49 | 0.38 | 0.56 | 0.98 | 0.56 | 0.42 | 0.57 |
| *Eubacterium*_g8 | 0.74 | 0.39 | 0.35 | 0.53 | 0.71 | 0.39 | 0.32 | 0.55 | 0.79 | 0.42 | 0.37 | 0.53 |
| *Ruminococcus*_g2 | 0.87 | 0.46 | 0.41 | 0.53 | 0.85 | 0.47 | 0.38 | 0.55 | 0.97 | 0.33 | 0.64 | 0.34 |
| UC_Christensenellaceae (PAC001207_g) | 1.03 | 0.54 | 0.49 | 0.52 | 0.96 | 0.55 | 0.41 | 0.58 | 1.31 | 0.70 | 0.61 | 0.53 |
| *Ruminococcus* | 1.05 | 0.55 | 0.50 | 0.52 | 1.06 | 0.60 | 0.46 | 0.57 | 1.00 | 0.58 | 0.42 | 0.58 |
| UC_Christensenellaceae (PAC001360_g) | 0.78 | 0.36 | 0.42 | 0.46 | 0.78 | 0.41 | 0.37 | 0.52 | 0.73 | 0.23 | 0.50 | 0.31 |
| *Eubacterium*_g23 | 0.93 | 0.41 | 0.52 | 0.44 | 0.81 | 0.35 | 0.46 | 0.44 | 1.12 | 0.45 | 0.67 | 0.40 |
| *Caproiciproducens* | 0.72 | 0.29 | 0.43 | 0.41 | 0.74 | 0.31 | 0.43 | 0.42 | 0.75 | 0.36 | 0.39 | 0.48 |
| *Lactobacillus* | 0.95 | 0.38 | 0.57 | 0.40 | 0.82 | 0.40 | 0.42 | 0.49 | 1.39 | 0.46 | 0.93 | 0.33 |
| *Odoribacter* | 0.82 | 0.31 | 0.51 | 0.38 | 0.78 | 0.30 | 0.48 | 0.39 | 1.02 | 0.51 | 0.51 | 0.50 |
| UC_Ruminococcaceae | 0.64 | 0.24 | 0.40 | 0.38 | 0.55 | 0.17 | 0.38 | 0.31 | 0.80 | 0.39 | 0.41 | 0.48 |
| *Coprococcus*_g2 | 0.63 | 0.22 | 0.41 | 0.36 | 0.56 | 0.10 | 0.46 | 0.18 | 0.62 | 0.49 | 0.13 | 0.79 |
| UC_Lachnospiraceae (PAC001043_g) | 0.71 | 0.26 | 0.45 | 0.36 | 0.67 | 0.22 | 0.45 | 0.33 | 0.61 | 0.00 | 0.61 | 0.00 |
| *Prevotella* | 0.60 | 0.21 | 0.39 | 0.35 | 0.43 | 0.16 | 0.27 | 0.37 | 1.05 | 0.47 | 0.58 | 0.44 |
| *Eubacterium*_g24 | 1.31 | 0.46 | 0.85 | 0.35 | 1.32 | 0.44 | 0.88 | 0.33 | 1.38 | 0.53 | 0.85 | 0.39 |
| *Oscillibacter* | 0.63 | 0.22 | 0.41 | 0.35 | 0.62 | 0.23 | 0.39 | 0.37 | 0.60 | 0.19 | 0.41 | 0.31 |
| UC_Erysipelotrichaceae (CCMM_g) | 0.92 | 0.32 | 0.60 | 0.35 | 0.92 | 0.36 | 0.56 | 0.39 | 0.77 | 0.15 | 0.62 | 0.19 |
| *Collinsella* | 0.85 | 0.29 | 0.56 | 0.34 | 0.84 | 0.35 | 0.49 | 0.42 | 0.97 | 0.34 | 0.63 | 0.35 |
| *Clostridium* | 1.15 | 0.39 | 0.76 | 0.34 | 1.09 | 0.30 | 0.79 | 0.27 | 1.47 | 1.01 | 0.46 | 0.69 |
| UC_Christensenellaceae (PAC001115_g) | 0.91 | 0.30 | 0.61 | 0.33 | 0.91 | 0.30 | 0.61 | 0.33 | 0.88 | 0.39 | 0.49 | 0.44 |
| UC_Ruminococcaceae (PAC000672_g) | 0.80 | 0.26 | 0.54 | 0.33 | 0.74 | 0.25 | 0.49 | 0.33 | 0.98 | 0.32 | 0.66 | 0.32 |
| *Clostridium*_g24 | 0.91 | 0.29 | 0.62 | 0.32 | 0.87 | 0.26 | 0.61 | 0.30 | 1.03 | 0.63 | 0.40 | 0.61 |
| *Bacteroides* | 0.19 | 0.06 | 0.13 | 0.31 | 0.18 | 0.06 | 0.12 | 0.31 | 0.18 | 0.07 | 0.11 | 0.39 |
| *Frisingicoccus* | 0.55 | 0.16 | 0.39 | 0.30 | 0.48 | 0.15 | 0.33 | 0.31 | 0.71 | 0.30 | 0.41 | 0.42 |
| *Anaerostipes* | 0.88 | 0.26 | 0.62 | 0.29 | 0.91 | 0.26 | 0.65 | 0.28 | 0.88 | 0.41 | 0.47 | 0.47 |
| *Bifidobacterium* | 0.98 | 0.25 | 0.73 | 0.26 | 0.95 | 0.30 | 0.65 | 0.32 | 1.15 | 0.27 | 0.88 | 0.24 |
| *Streptococcus* | 0.77 | 0.16 | 0.61 | 0.20 | 0.75 | 0.29 | 0.46 | 0.39 | 0.75 | 0.03 | 0.72 | 0.04 |
| *Veillonella* | 0.55 | 0.11 | 0.44 | 0.20 | 0.52 | 0.10 | 0.42 | 0.19 | 0.66 | 0.10 | 0.56 | 0.15 |
| *Lachnospira* | 0.59 | 0.11 | 0.48 | 0.19 | 0.56 | 0.13 | 0.43 | 0.24 | 0.71 | 0.00 | 0.71 | 0.01 |
| *Parabacteroides* | 0.34 | 0.06 | 0.28 | 0.18 | 0.37 | 0.06 | 0.31 | 0.17 | 0.17 | 0.03 | 0.14 | 0.19 |
| *Anaerotignum* | 0.60 | 0.11 | 0.49 | 0.18 | 0.58 | 0.08 | 0.50 | 0.13 | 0.66 | 0.27 | 0.39 | 0.41 |
| UC_Peptostreptococcaceae | 0.79 | 0.13 | 0.66 | 0.17 | 0.77 | 0.10 | 0.67 | 0.13 | 0.84 | 0.20 | 0.64 | 0.24 |
| *Escherichia* | 1.04 | 0.17 | 0.87 | 0.16 | 1.01 | 0.17 | 0.84 | 0.17 | 1.07 | 0.04 | 1.03 | 0.04 |
| *Haemophilus* | 0.89 | 0.14 | 0.75 | 0.16 | 0.90 | 0.18 | 0.72 | 0.20 | 0.84 | 0.00 | 0.84 | 0.00 |
| UC_Lachnospiraceae | 0.04 | 0.00 | 0.04 | 0.12 | 0.04 | 0.01 | 0.03 | 0.18 | 0.04 | 0.00 | 0.04 | 0.00 |
| *Romboutsia* | 0.75 | 0.09 | 0.66 | 0.12 | 0.76 | 0.05 | 0.71 | 0.06 | 0.80 | 0.53 | 0.27 | 0.66 |
| *Blautia* | 0.09 | 0.01 | 0.08 | 0.11 | 0.08 | 0.01 | 0.07 | 0.06 | 0.11 | 0.01 | 0.10 | 0.10 |
| *Pseudoflavonifractor* | 0.44 | 0.04 | 0.40 | 0.10 | 0.54 | 0.11 | 0.43 | 0.21 | 0.16 | 0.01 | 0.15 | 0.07 |
| *Roseburia* | 0.66 | 0.06 | 0.60 | 0.09 | 0.68 | 0.16 | 0.52 | 0.23 | 0.58 | 0.00 | 0.58 | 0.00 |
| *Ruminococcus*_g4 | 0.47 | 0.02 | 0.45 | 0.05 | 0.51 | 0.01 | 0.50 | 0.02 | 0.28 | 0.13 | 0.15 | 0.45 |
| *Faecalibacterium* | 0.10 | 0.00 | 0.10 | 0.05 | 0.05 | 0.01 | 0.04 | 0.20 | 0.27 | 0.02 | 0.25 | 0.08 |
| *Eubacterium*_g5 | 0.66 | 0.02 | 0.64 | 0.03 | 0.69 | 0.01 | 0.68 | 0.01 | 0.38 | 0.09 | 0.29 | 0.24 |
| UC_Lachnospiraceae (PAC001138_g) | 0.76 | 0.02 | 0.74 | 0.03 | 0.78 | 0.06 | 0.72 | 0.08 | 0.75 | 0.00 | 0.75 | 0.00 |
| *Agathobaculum* | 0.69 | 0.02 | 0.67 | 0.03 | 0.69 | 0.04 | 0.65 | 0.06 | 0.71 | 0.00 | 0.71 | 0.00 |

**Supplementary Table 5.** Inter- and intra-individual variances of dominant genera in the M3 type gut microbiota across time points. Dominant genera were selected by median abundance ≥ 0.1% and prevalence ≥ 30% in the M3 gut microbiota. Variances were calculated at each time point. UC, unclassified; DS, During Stay which included HOME and BASE samples; AL, After Leaving which included SHIP and RETURN samples; HOME, before participants departed from Korea; BASE, stay at Antarctic stations; SHIP, ship voyage after leaving the stations; RETURN, after returning to Korea; ICC, intra-class correlation coefficient.

| Genus | All period | | | ICC | DS | | | ICC | AL | | | ICC |
| --- | --- | --- | --- | --- | --- | --- | --- | --- | --- | --- | --- | --- |
|  | variance in abundance, Total | Inter-individual variance (τ2) | Intra-individual variance (α2) |  | variance in abundance, Total | Inter-individual variance (τ2) | Intra-individual variance (α2) |  | variance in abundance, Total | Inter-individual variance (τ2) | Intra-individual variance (α2) |  |
| *Megamonas* | 2.33 | 2.31 | 0.02 | 0.99 | 2.30 | 2.28 | 0.02 | 0.99 | 2.48 | 2.47 | 0.01 | 0.99 |
| *Prevotella* | 2.69 | 2.41 | 0.28 | 0.90 | 2.86 | 2.66 | 0.20 | 0.93 | 2.80 | 2.53 | 0.27 | 0.90 |
| *Ruminococcus*_g4 | 1.05 | 0.84 | 0.21 | 0.80 | 1.05 | 0.81 | 0.24 | 0.77 | 1.15 | 0.91 | 0.24 | 0.79 |
| UC_Erysipelotrichaceae | 1.46 | 1.15 | 0.31 | 0.79 | 1.36 | 1.11 | 0.25 | 0.81 | 1.70 | 1.41 | 0.29 | 0.83 |
| *Phascolarctobacterium* | 1.22 | 0.94 | 0.28 | 0.77 | 1.22 | 0.94 | 0.28 | 0.77 | 1.20 | 0.93 | 0.27 | 0.77 |
| *Dialister* | 1.20 | 0.82 | 0.38 | 0.69 | 1.19 | 0.85 | 0.34 | 0.71 | 1.32 | 0.90 | 0.42 | 0.68 |
| *Bacteroides* | 0.15 | 0.10 | 0.05 | 0.67 | 0.12 | 0.08 | 0.04 | 0.69 | 0.19 | 0.15 | 0.04 | 0.77 |
| UC_Erysipelotrichaceae (CCMM_g) | 0.89 | 0.59 | 0.30 | 0.66 | 0.84 | 0.58 | 0.26 | 0.69 | 0.93 | 0.57 | 0.36 | 0.61 |
| *Barnesiella* | 1.06 | 0.68 | 0.38 | 0.64 | 1.03 | 0.68 | 0.35 | 0.66 | 1.06 | 0.63 | 0.43 | 0.59 |
| *Eubacterium*_g5 | 0.60 | 0.39 | 0.21 | 0.64 | 0.76 | 0.55 | 0.21 | 0.72 | 0.45 | 0.18 | 0.27 | 0.40 |
| *Coprobacter* | 0.61 | 0.35 | 0.26 | 0.58 | 0.55 | 0.34 | 0.21 | 0.62 | 0.76 | 0.39 | 0.37 | 0.51 |
| UC_Ruminococcaceae (PAC000661_g) | 1.42 | 0.77 | 0.65 | 0.54 | 1.43 | 0.67 | 0.76 | 0.47 | 1.19 | 0.60 | 0.59 | 0.51 |
| *Coprococcus*_g2 | 0.87 | 0.46 | 0.41 | 0.53 | 0.95 | 0.53 | 0.42 | 0.56 | 0.88 | 0.65 | 0.23 | 0.74 |
| *Mitsuokella* | 0.95 | 0.50 | 0.45 | 0.53 | 0.82 | 0.38 | 0.44 | 0.47 | 0.96 | 0.31 | 0.65 | 0.32 |
| *Butyricimonas* | 0.92 | 0.48 | 0.44 | 0.52 | 0.84 | 0.38 | 0.46 | 0.46 | 0.89 | 0.16 | 0.73 | 0.18 |
| *Anaerostipes* | 0.41 | 0.21 | 0.20 | 0.51 | 0.34 | 0.11 | 0.23 | 0.34 | 0.30 | 0.07 | 0.23 | 0.24 |
| *Ruminococcus*_g2 | 0.95 | 0.44 | 0.51 | 0.46 | 1.00 | 0.46 | 0.54 | 0.46 | 1.04 | 0.49 | 0.55 | 0.47 |
| UC_Peptostreptococcaceae | 0.95 | 0.38 | 0.57 | 0.40 | 0.95 | 0.38 | 0.57 | 0.40 | 0.80 | 0.13 | 0.67 | 0.16 |
| *Collinsella* | 0.61 | 0.23 | 0.38 | 0.38 | 0.67 | 0.29 | 0.38 | 0.43 | 0.87 | 0.22 | 0.65 | 0.25 |
| UC_Ruminococcaceae (PAC000748_g) | 0.69 | 0.26 | 0.43 | 0.38 | 0.60 | 0.23 | 0.37 | 0.38 | 0.74 | 0.23 | 0.51 | 0.31 |
| *Clostridium*_g21 | 0.86 | 0.29 | 0.57 | 0.34 | 0.87 | 0.27 | 0.60 | 0.31 | 0.76 | 0.17 | 0.59 | 0.22 |
| *Eubacterium*_g23 | 0.67 | 0.23 | 0.44 | 0.34 | 0.72 | 0.18 | 0.54 | 0.25 | 0.48 | 0.23 | 0.25 | 0.47 |
| *Parabacteroides* | 0.14 | 0.05 | 0.09 | 0.33 | 0.10 | 0.03 | 0.07 | 0.32 | 0.19 | 0.08 | 0.11 | 0.44 |
| *Eubacterium*_g24 | 1.20 | 0.36 | 0.84 | 0.30 | 1.27 | 0.39 | 0.88 | 0.31 | 1.03 | 0.41 | 0.62 | 0.40 |
| *Sporobacter* | 0.60 | 0.18 | 0.42 | 0.30 | 0.59 | 0.15 | 0.44 | 0.25 | 0.60 | 0.09 | 0.51 | 0.14 |
| UC_Ruminococcaceae (PAC000672_g) | 0.60 | 0.16 | 0.44 | 0.27 | 0.65 | 0.19 | 0.46 | 0.30 | 0.62 | 0.09 | 0.53 | 0.15 |
| UC_Lachnospiraceae (PAC001138_g) | 0.95 | 0.25 | 0.70 | 0.26 | 0.99 | 0.21 | 0.78 | 0.21 | 0.85 | 0.50 | 0.35 | 0.59 |
| *Ruminococcus* | 0.79 | 0.20 | 0.59 | 0.25 | 0.71 | 0.09 | 0.62 | 0.13 | 0.81 | 0.18 | 0.63 | 0.22 |
| *Desulfovibrio* | 0.62 | 0.15 | 0.47 | 0.24 | 0.61 | 0.18 | 0.43 | 0.29 | 0.69 | 0.13 | 0.56 | 0.19 |
| *Frisingicoccus* | 0.53 | 0.12 | 0.41 | 0.22 | 0.53 | 0.03 | 0.50 | 0.05 | 0.45 | 0.31 | 0.14 | 0.69 |
| *Caproiciproducens* | 0.65 | 0.14 | 0.51 | 0.22 | 0.64 | 0.12 | 0.52 | 0.18 | 0.64 | 0.20 | 0.44 | 0.31 |
| *Clostridium*_g24 | 0.87 | 0.18 | 0.69 | 0.20 | 0.82 | 0.13 | 0.69 | 0.16 | 0.92 | 0.28 | 0.64 | 0.30 |
| UC_Ruminococcaceae | 0.44 | 0.09 | 0.35 | 0.20 | 0.44 | 0.09 | 0.35 | 0.20 | 0.50 | 0.07 | 0.43 | 0.14 |
| *Alistipes* | 0.55 | 0.10 | 0.45 | 0.19 | 0.45 | 0.07 | 0.38 | 0.16 | 0.76 | 0.17 | 0.59 | 0.22 |
| *Bifidobacterium* | 0.44 | 0.08 | 0.36 | 0.18 | 0.26 | 0.12 | 0.14 | 0.47 | 0.66 | 0.00 | 0.66 | 0.00 |
| UC_Christensenellaceae (PAC001207_g) | 0.68 | 0.12 | 0.56 | 0.18 | 0.63 | 0.07 | 0.56 | 0.11 | 0.79 | 0.17 | 0.62 | 0.21 |
| *Anaerotignum* | 0.60 | 0.10 | 0.50 | 0.17 | 0.54 | 0.11 | 0.43 | 0.21 | 0.66 | 0.01 | 0.65 | 0.02 |
| *Blautia* | 0.05 | 0.01 | 0.04 | 0.17 | 0.05 | 0.00 | 0.05 | 0.10 | 0.04 | 0.01 | 0.03 | 0.23 |
| *Romboutsia* | 0.90 | 0.15 | 0.75 | 0.17 | 0.89 | 0.36 | 0.53 | 0.40 | 1.03 | 0.13 | 0.90 | 0.12 |
| *Turicibacter* | 0.67 | 0.11 | 0.56 | 0.16 | 0.52 | 0.03 | 0.49 | 0.06 | 0.80 | 0.10 | 0.70 | 0.13 |
| *Haemophilus* | 0.97 | 0.16 | 0.81 | 0.16 | 1.00 | 0.13 | 0.87 | 0.13 | 0.83 | 0.25 | 0.58 | 0.30 |
| *Streptococcus* | 0.74 | 0.11 | 0.63 | 0.15 | 0.72 | 0.11 | 0.61 | 0.15 | 0.72 | 0.23 | 0.49 | 0.33 |
| *Lachnospira* | 0.15 | 0.02 | 0.13 | 0.15 | 0.13 | 0.03 | 0.10 | 0.26 | 0.15 | 0.01 | 0.14 | 0.09 |
| *Faecalibacterium* | 0.06 | 0.01 | 0.05 | 0.15 | 0.05 | 0.00 | 0.05 | 0.00 | 0.06 | 0.03 | 0.03 | 0.48 |
| *Pseudoflavonifractor* | 0.49 | 0.07 | 0.42 | 0.15 | 0.50 | 0.13 | 0.37 | 0.26 | 0.58 | 0.01 | 0.57 | 0.02 |
| UC_Lachnospiraceae (PAC001043_g) | 0.66 | 0.09 | 0.57 | 0.13 | 0.70 | 0.06 | 0.64 | 0.08 | 0.50 | 0.26 | 0.24 | 0.52 |
| UC_Lachnospiraceae | 0.02 | 0.00 | 0.02 | 0.10 | 0.02 | 0.00 | 0.02 | 0.00 | 0.03 | 0.02 | 0.01 | 0.60 |
| *Roseburia* | 0.48 | 0.04 | 0.44 | 0.09 | 0.50 | 0.07 | 0.43 | 0.14 | 0.45 | 0.03 | 0.42 | 0.07 |
| *Escherichia* | 1.08 | 0.08 | 1.00 | 0.08 | 1.08 | 0.02 | 1.06 | 0.02 | 1.12 | 0.03 | 1.09 | 0.02 |
| *Agathobaculum* | 0.65 | 0.05 | 0.60 | 0.07 | 0.66 | 0.02 | 0.64 | 0.03 | 0.78 | 0.14 | 0.64 | 0.18 |
| *Sutterella* | 0.51 | 0.04 | 0.47 | 0.07 | 0.45 | 0.05 | 0.40 | 0.12 | 0.60 | 0.00 | 0.60 | 0.00 |
| *Subdoligranulum* | 0.17 | 0.01 | 0.16 | 0.06 | 0.13 | 0.00 | 0.13 | 0.03 | 0.20 | 0.03 | 0.17 | 0.13 |
| *Oscillibacter* | 0.28 | 0.01 | 0.27 | 0.02 | 0.21 | 0.00 | 0.21 | 0.00 | 0.51 | 0.00 | 0.51 | 0.00 |
| *Odoribacter* | 0.58 | 0.00 | 0.58 | 0.00 | 0.54 | 0.03 | 0.51 | 0.05 | 0.75 | 0.00 | 0.75 | 0.00 |
| *Clostridium* | 0.87 | 0.00 | 0.87 | 0.00 | 0.88 | 0.00 | 0.88 | 0.00 | 0.72 | 0.00 | 0.72 | 0.00 |
